# Enhancing and Extending the Meta-Analytic Comparison of Newer Genre Leadership Forms

**DOI:** 10.3389/fpsyg.2022.872568

**Published:** 2022-04-12

**Authors:** Bryan Fuller, Abdulah Bajaba, Saleh Bajaba

**Affiliations:** ^1^Department of Management, Louisiana Tech University, Ruston, LA, United States; ^2^Department of Business Administration, King Abdulaziz University, Jeddah, Saudi Arabia

**Keywords:** ethical leadership, servant leadership, authentic leadership, transformational leadership, meta-analysis

## Abstract

Interest in leadership research is growing, however, the rate of leadership learning is slowing down due to the proliferation of new leadership constructs. The objective of the present meta-analysis is to address the significant shortcomings in prior meta-analytic research on newer genre leadership forms by (a) utilizing a substantially greater number of studies and observations than in previous meta-analyses and (b) examining the meta-analytic correlations among the newer genre leadership forms. The results of the present study indicate that the newer genre leadership forms overlap to a greater degree than previously reported, while at the same time accounting for some degree of unique variance in the literature's most studied outcome variables; estimates of the relative contribution of each leadership form to the outcomes are provided, providing new insights into the distinctiveness of each leadership form. The findings suggest that pursuing an integrated theory and measure of newer genre leadership forms is a desirable future step for leadership research.

## Introduction

The interest in leadership and leadership development among scholars and practitioners has been growing exponentially in the last two decades (Hopkins and O'Neil, 2015; Sarid, 2016; Jiang et al., 2017; Xenikou, 2017; Legutko, 2020; Legood et al., 2021; Li et al., 2021; Blake et al., 2022); such interest has spawned so many new leadership constructs, thereby, raising concerns regarding the effect of such a consequence on the advancement of the leadership literature (Meuser et al., 2016; Antonakis, 2017; Rodriguez et al., 2017; Banks et al., 2018; Hoch et al., 2018). That is, the rate of learning may be actually slowing down because little is known about the extent to which the plethora of leadership constructs offer unique insights into leadership behavior or the leadership process. The problem of overlapping leadership constructs, particularly, the newer genre leadership forms (NGL; transformational, authentic, ethical, and servant leadership; Hannah et al., 2014), has caused confusion about how to move the field forward. Some literature recommendations argued that the best way forward is to launch research programs aimed at developing better theory and instrumentation for those NGL forms, based upon the belief that it will result in greater discriminant validity and incremental contribution for each leadership form (e.g., Hannah et al., 2014; Banks et al., 2016; Avolio et al., 2018; Eva et al., 2019; Lemoine et al., 2019; Lee et al., 2020). In contrast, other literature recommendations called for a parsimony-oriented approach, integrating existing leadership theories where warranted, thereby reducing the number of leadership theories (e.g., Dinh et al., 2014; Meuser et al., 2016; Banks et al., 2018).

Recent meta-analytic reviews compared the NGL theories and have attempted to resolve some of the confusion about this particular domain of leadership (i.e., Banks et al., 2016, 2018; Hoch et al., 2018). Conclusions drawn from these efforts include the following: (a) transformational, authentic, and ethical leadership are largely redundant, but servant leadership appears to be distinct from these forms of leadership (Hoch et al., 2018; Lee et al., 2020), (b) authentic and transformational leadership are so highly correlated suggesting redundancy, yet research on authentic leadership should continue by focusing on a better theory, measurement, and research design to differentiate it from transformational leadership (Banks et al., 2016), (c) of the four NGL forms, transformational leadership is the best predictor of job performance (Banks et al., 2018), and (d) transformational, authentic, ethical, and servant leadership should all be integrated into one grand unified theory of leadership, and a corresponding measurement instrument should be developed (Banks et al., 2018). Overall, there is a lack of agreement about what the best path forward is at this point.

The purpose of the present research is to provide a more solid foundation upon which to make decisions about the most fruitful directions for future leadership research, and it is done by addressing the major shortcomings of the previous meta-analytic research. One shortcoming of prior meta-analytic research is the lack of sufficient studies to provide a high degree of confidence in the stability of the reported effect size estimates (Hoch et al., 2018; Eva et al., 2019). The analyses in this study are based upon more than 2.5 times the average number of studies and observations used in previous meta-analyses, thus, contributing to the literature by providing much greater confidence in the effect size estimates for the relationships examined in the literature. The second shortcoming is striking given the primary purpose of the previous meta-analyses—only Lee et al. (2020) of the recent meta-analytic studies included a meta-analytic aggregation of studies to estimate the magnitude of the intercorrelations between authentic, ethical, and servant leadership, that being said, with a limited number of studies. This limitation reduces the accuracy/ability of previous meta-analytic research to account for omitted-variable-based endogeneity bias (Antonakis, 2017) and, thus, precludes the understanding of the true effects of each leadership form (Eva et al., 2019). The current study addresses this by providing the meta-analytically derived effect size estimates missing in prior research (i.e., the intercorrelations among authentic, ethical, and servant leadership forms) with a much larger sample of studies, resulting in more accurate relative weight analyses that include all four leadership forms. By doing so, the current study provides accurate estimates of the relative contribution that each leadership form makes to some of the most studied outcomes in the extant literature (i.e., leader-member exchange or LMX, organizational commitment, job satisfaction, engagement, intention to leave, and trust in leader) while providing a more accurate reflection of others (i.e., job performance, organizational citizenship behavior or OCB, and counterproductive work behavior or CWB). In short, the major contribution of the present research is providing a much more solid foundation for concluding the distinctiveness and contribution of the four NGL forms, which will better inform the scholars' decisions about directions for future research in the leadership literature.

## Theoretical Background

In this section, the definitions and dimensions of the four NGL forms are briefly reviewed to summarize the conceptual foundations of each form. Then, the NGL forms are compared in terms of their commonalities as a prelude to the empirical examination.

### Transformational Leadership

Since its conceptualization in the early 1980s, transformational leadership (TL) has been one of the most studied leadership theories over the past few decades (Mhatre and Riggio, 2014) and has been frequently compared with transactional leadership (Burns, 1978). Whereas, transactional leadership purpose was short-term self-interest and sole exchange of values between the leader and the follower, TL was based on the leader engaging and creating a connection with the followers to increase motivation and morality, and thus, transforming both the leader and follower (Burns, 1978; Northouse, 2018). Future work built upon this idea of the transactional-transformational model of leadership by developing a new continuum known as the full range of leadership model that had three elements ranging from TL to transactional leadership, and ultimately, absence of leadership (or laissez-faire; Bass, 1985).

TL was defined as a leadership form that aims to “achieve follower performance beyond ordinary limits” (xiii) and was proposed to be represented by four dimensions: idealized influence, inspirational motivation, intellectual stimulation, and individualized consideration (Bass, 1985). Idealized influence refers to the leader's ability to serve as a positive model for the follower through high moral standards and ethical conduct, which conveys an ideological vision to the follower along with a sense of collective purpose (Bass, 1998; Bass and Riggio, 2006; Mhatre and Riggio, 2014). Inspirational motivation refers to the leader's ability to inspire and motivate followers by visioning high-performance expectations while simultaneously boosting their confidence to reach them (Bass, 1998; Bass and Riggio, 2006; Mhatre and Riggio, 2014). Intellectual stimulation refers to the leader's ability to stimulate followers' creativity and innovation through challenging tasks as the leader conveys to the follower that they are empowered and trusted. Lastly, individualized consideration refers to the leader's ability to be responsive to the followers' needs and to be able to provide guidance, supports, and mentorship to further enhance their performance and potential (Bass, 1998; Bass and Riggio, 2006; Mhatre and Riggio, 2014).

### Authentic Leadership

The theory of authentic leadership (AL) was ignited with the conceptualization of Luthans and Avolio (2003) where AL was viewed as a positive form of leadership instead of negative or inauthentic (Seeman, 1960). Such conceptualization stimulated further research efforts to develop AL from an organizational perspective (Avolio et al., 2004a; Avolio and Gardner, 2005; Gardner et al., 2005). Authentic leaders were defined as those who have a high sense of awareness when it comes to their thinking and behavior and are perceived by themselves and others as being so; moreover, they are fully aware of the context they operate in and have high levels of confidence, optimism, hope, resiliency, and moral character (Avolio et al., 2004b). The definition of AL was further refined to reflect a set of leader behaviors that emphasize both positive psychological capacities and positive ethical climate to foster greater leader self-awareness, internalized moral perspective, balanced processing of information, and relational transparency when working with followers, thereby, fostering positive self-development (Walumbwa et al., 2008, p. 94); the above definition reflects the four dimensions of AL: self-awareness, relational transparency, balanced processing, and internalized moral perspective.

The self-awareness dimension of AL refers to the leader's ability to be aware of their own emotions, values, goals, weaknesses, and strengths (Gardner et al., 2005; Ilies et al., 2013). Moreover, it includes insight or knowledge of “one's multifaceted nature of the self,” which can be gained through exposure to others and being aware of one's impact on them (Walumbwa et al., 2008, p. 95). Relational transparency refers to the leader's transparency in sharing information and expressing their true emotions and thoughts to others without faking or distortion (Walumbwa et al., 2008). An authentic leader is shown to others in an open, honest, yet appropriate manner, which promotes trust in relationships (Kernis, 2003; Wong and Cummings, 2009; Zhang et al., 2021). Balanced processing is a dimension that reflects the leader's ability to keep an objective mindset when making decisions while simultaneously considering others' opinions and thoughts (Walumbwa et al., 2008). Lastly, internalized moral perspective refers to the leader being able to self-regulate through internal moral standards that are not affected by group, organizational, or societal pressures (Walumbwa et al., 2008). In sum, authentic leaders can make decisions consistent with their internal values and moral standards (Walumbwa et al., 2008). The work of Walumbwa et al. (2008) is only one out of many other efforts to conceptualize AL (see Gardner et al., 2011 for a review).

### Ethical Leadership

Public corporate scandals over the past decade, such as Enron, WorldCom, Nortel, AIG, and Lehman Brothers, have directed a high amount of attention toward the ethicality of corporate decision-making and leadership. Ethical leadership was defined as personal and interpersonal actions that demonstrate normatively appropriate conduct and promote such conduct to followers through two-way communication, reinforcement, and decision-making (Brown et al., 2005, p. 120). In other words, an ethical leader is a leader who is fair, honest, trustworthy, and principled decision-makers that behaves ethically in his/her personal and professional lives (Brown and Treviño, 2006; Charoensap et al., 2019). Moreover, ethical leaders do not only play the role of moral people by behaving ethically, but they also become moral managers such that they act as role models to influence followers to behave ethically as well (Brown and Treviño, 2006; O'Keefe et al., 2019). Two theories can rationalize how ethical leaders influence the followers' ethical behavior and standards: social learning theory (Bandura, 1977) and social exchange theory (Blau, 1964).

Social learning theory refers to the idea that individuals pay attention to what is attractive in the environment, such as context and other individuals, for guidance (Bandura, 1977). Ethical leaders act as attractive role models to learn from due to their fairness, credibility, and trustworthiness (Brown and Treviño, 2006). Consequently, followers pay attention to the behaviors of the ethical leader to understand what is normatively acceptable and what is not. Moreover, learning gets reinforced when the leader applies rewards and punishments to influence ethical behavior (Brown and Treviño, 2006). Social exchange theory states that social exchange tends to result in feelings of gratitude, trust, and personal obligation, which is different from the transactional exchange as they are contract-like (Blau, 1964, p. 94; Homans, 1961). Therefore, due to the fairness, honesty, and caring of the ethical leader, the followers of the ethical leader are more likely to perceive themselves in a social exchange or personal obligation to reciprocate and “go above and beyond the call of duty for these leaders” (Brown and Treviño, 2006, p. 607). To summarize, ethical leaders tend to influence the followers' ethical behaviors *directly* through role modeling and *indirectly* through creating an environment of reciprocity and social exchange (Bedi et al., 2016).

### Servant Leadership

Although servant leadership (SL) was introduced earlier than TL (Greenleaf, 1970), it had not attracted much attention until recently (Liden et al., 2008; Dinh et al., 2014; Lumpkin and Achen, 2018; Eva et al., 2019). Greenleaf's (1970) proposed that servant leaders' highest priority is serving the others' needs and are stimulated by a natural feeling that turns into a conscious choice of leading through serving: “the servant-leader is servant first” (p. 13). Since then, numerous researchers have built upon Greenleaf's (1970, 1977) work (Liden et al., 2008; Spears, 2010; van Dierendonck and Nuijten, 2011). Servant leaders have goals that are not based on self-interest as they aim to form long-term relationships with their followers that extend outside the organization instead of treating organizational goals as their end goal (Ehrhart, 2004; Liden et al., 2008). Eight dimensions were proposed for servant leadership: emotional healing, creating value for the community, conceptual skills, empowering, helping subordinates grow and succeed, putting subordinates first, behaving ethically (Liden et al., 2008).

Although Greenleaf's (1970) definition of SL has been consistently referenced (e.g., van Dierendonck, 2011), it defined SL in terms of its outcomes instead of its behaviors, thus, affecting its utility as a construct definition (Lemoine et al., 2019). Therefore, more recent efforts proposed a refined definition (Eva et al., 2019): an other-oriented approach to leadership that prioritizes the others' individual needs and interests within the organization and the larger community (p. 114). Furthermore, three features of SL were emphasized: motive, mode, and mindset (Eva et al., 2019). The motive aspect indicates that a servant leader has the personal motivation to take responsibility for others instead of focusing on advancing their own agenda or ambitions. The mode aspect emphasizes that the relationship between a servant leader and each follower should be unique, reflecting each follower's different needs, desires, interests, and goals. Lastly, the mindset aspect refers to the servant leader's deliberate focus on follower development, becoming servant leaders (Eva et al., 2019).

### The Overlap Between the Four Newer Genre Leadership Forms

The problematic overlap between the NGL forms has been frequently emphasized in the leadership literature (e.g., Anderson and Sun, 2017; Banks et al., 2018; Lemoine et al., 2019). For instance, TL has been suggested to overlap with SL as they both emphasize the need for vision and influence (Bass, 2000, p. 33) by encouraging others to visualize the organization's future and offering compelling reasons to take action (Barbuto and Wheeler, 2006, p. 319). Although some literature argue that the prosocial vision of SL is different from that of the organizational vision of TL (Stone et al., 2004; van Dierendonck, 2011), other efforts propose that TL can be prosocial as well, thus, benefitting the members of the organization alongside meeting the organization's goals (Grant, 2012).

AL and TL appear to overlap a great deal as well. Multiple areas of shared aspects between the two theories, such as a leader's self-awareness and regulation processes, were discussed (Avolio and Gardner, 2005). What made AL unique as a leadership theory was that it promoted positive organizational behavior states of optimism, resiliency, and hope (Avolio and Gardner, 2005; Anderson and Sun, 2017). However, recent research indicates that these psychological resources are related to both TL and AL (Peterson et al., 2009; Gardner et al., 2011). Until recently, the relationship between these two leadership theories has been ambiguous and not adequately distinguished over the years (Neider and Schriesheim, 2011) as AL is sometimes measured using TL items from the MLQ scale (Jensen and Luthans, 2006).

In addition, EL has some key differences from other leadership styles, such as TL and AL (Brown and Treviño, 2006). For instance, the focus of EL is moral management and *other* awareness; on the other hand, TL emphasizes vision and intellectual stimulation, whereas AL emphasizes self-awareness (Brown and Treviño, 2006). However, key similarities between these leadership styles were also discussed: role modeling, integrity, ethical decision-making, and altruism (Brown and Treviño, 2006). Furthermore, transformational leaders require a strong moral foundation that are central to EL (Bass and Steidlmeier, 1999). AL also shares commonalities with EL regarding principles and ethics (Begley, 2001; Gardner et al., 2011). For example, one of the defining characteristics of authentic leaders is that they “lead from conviction in pursuit of a value-based mission or cause” (Shamir and Eilam, 2005, p. 397). Simply put, authentic leaders tend to have a deep sense of self, values, and beliefs, which they convey to others in terms of principles and ethics (Avolio and Gardner, 2005).

SL has also been linked to AL as authentic leaders try to serve others more effectively by showing a genuine desire to understand their own leadership behaviors, which is similar to the aspect of serving others in SL (Anderson and Sun, 2017, p. 96). Furthermore, a recent review of the empirical body of the moral leadership forms (i.e., servant, ethical, and authentic) by Lemoine et al. (2019) found more commonalities than differences between the constructs, represented by the prediction of expected outcomes, the utilization of common theories, and the investigation of common composition (or measurement overlap). For instance, servant and ethical leaders are suggested to show concern for followers, servant and authentic leaders are suggested to enhance followers' personal growth, and ethical and authentic leaders are suggested to have moral consistency (Lemoine et al., 2019). Moreover, all three moral leadership forms are suggested to demonstrate some form of moral/ethical behaviors (Lemoine et al., 2019).

In sum, although there might be some unique key differences between the NGL forms, the current state of the leadership literature and empirical body suggests a much larger overlap, which can be problematic (Banks et al., 2018; Lemoine et al., 2019). Therefore, the purpose of present analysis is to investigate the question of empirical redundancy and the overlap between the four NGL forms through an integrative meta-analytic examination based upon the theoretical commonalities discussed in this article and other works (e.g., Hoch et al., 2018; Lemoine et al., 2019) in order to offer insight for future directions in the leadership literature.

## Methods

### Literature Search

The studies for the present analyses were collected through a systematic computer-based search of the four leadership styles (authentic, servant, ethical, and transformational). Several databases and search engines were utilized in the process (e.g., *ABI-Inform Complete, ProQuest Research Library, ProQuest Digital Dissertations, JSTOR, Business Source Complete, Sage Journals, Google*, and *Google Scholar*). Manual searches were conducted of specific conference proceedings and programs such as the *Academy of Management Proceedings*. The following key words were used in the search for the studies of interest: servant leadership, ethical leadership, authentic leadership, transformational leadership, authentic, ethical, servant, transformational paired with correlation, and/or correlation matrix, and/or reliability. Reference lists of articles on these four leadership styles were also compared and searched through (i.e., Ng and Feldman, 2015; Banks et al., 2016, 2018; Hoch et al., 2018). The cutoff date for the collected studies was October 2018. Because of the number of studies and observations used in Hoch et al. (2018) analysis of the TL literature, no new meta-analytic aggregation of TL relationships was conducted as the number of studies and observations used in prior studies were sufficiently large to have confidence in the stability of the results (Hoch et al., 2018). That being said, an exception was made for (1) intention to leave (see Table 1), as this relationship has not been examined in prior meta-analytic research, and (2) the relationship between transformational leadership and the other three leadership forms, as the number of studies and observations in prior analyses in the literature were somewhat low and gathering additional studies for these relationships would be critical to the relative weight analyses.

**Table 1:** Comparison of current and prior meta-analytic results for transformational leadership and intention to leave.

**Variable**	***K***	***N***	$\bar{r}$	**ρ**	***SD*** **ρ**	**CI_**LL**_ CI_**UL**_**	***Z***
**Intention to leave**							
Current Study	17	7,360	−0.27	−0.31	0.01	(−0.36 −0.29)	
Without sample-size outliers	15	4,953	−0.30	−0.34	0.01	(−0.40 −0.32)	8.47*
Barlow (2013)	5	2,908	-	−0.31	-	(−0.34 −0.27)	0.00

K, number of studies; N, total sample size; r bar, average observed effect size; ρ, r bar corrected for measurement error in both the predictor and the criterion; SDρ, observed standard deviation in corrected correlations; CI_LL_ and CI_UL_, lower and upper bounds, respectively, of the 95% confidence interval around the mean true-score correlation; Z, Critical Ratio Z-score. A value of 1.64 or greater indicates a statistically significant difference (*p < 0.05) in mean true scores.

### Criteria for Inclusion

For the studies to be included in the meta-analysis, several established criteria had to be met. First, the studies had to have a working sample (including nurse samples and teacher-principal dyads)—non-working or student samples were excluded. Second, the studies had to report the sample size, the correlation between the leadership style and the variable of interest, and whether measures were self- or other-reports when applicable. Third, non-English studies were translated to English. Fourth, studies that were done only at the group level of analysis were excluded. For the purpose of consistency, only supervisor reports for job performance and OCB were used as supervisor reports tend to be the standard in leadership studies. On the other hand, only self-reports for CWB were used as this is the most common data collection method for this construct and the most consistent with the covert nature of the majority of these types of behavior.

### Coding Procedures

Two authors independently collected a subset of studies, and the overall list was revised by both authors for coding errors and discrepancies. The following information was collected on each study: scales used, their reliabilities, data source (multi/single source), sample size, and correlations of interest. For studies that did not report the reliability coefficient of the scales, an average of the scale reliabilities was computed following the steps of Banks et al. (2016). Furthermore, for studies that reported only the correlations of the related dimensions of the constructs without the overall one, an average of the dimension correlations was computed following the steps of Hoch et al. (2018).

### Meta-Analytic Procedures

Schmidt and Hunter (2014) random-effects model was used to conduct this meta-analysis due to its predominance in the literature and accuracy (Hunter and Schmidt, 1990, 2004; Hall and Brannick, 2002). This method allows the generation of sample-weighted estimates of the population criterion by correcting sampling errors and attenuation due to unreliability in the criterion and predictor (Fuller and Marler, 2009). In order to accurately interpret the results, a two-step process outlined by Whitener (1990) was utilized. The first step includes investigating whether moderators are present in the data. The second step utilizes the information from step 1 in order to measure the accuracy of the estimated population mean effect size. Based on that, it was determined whether the group of studies involved in this meta-analysis for each relationship measured was from one population (homogenous, indicating the absence of moderation) or from more than one population (heterogeneous, indicating the presence of unidentified moderators). Furthermore. the ubiquitous 75% rule was utilized to provide an indication of heterogeneity/homogeneity. This “omnibus” test is considered a better assessment of homogeneity than significance tests (Hunter and Schmidt, 2004). 95% credibility intervals were also calculated, as suggested by Whitener (1990), to provide another assessment for the presence of moderators (i.e., having zero in the interval or being too wide indicates heterogeneity).

Furthermore, 95% confidence intervals were calculated (Whitener, 1990) to account for the significance of the estimates mean effect size; confidence intervals provide a range of where the population means effect size can be located based on the observed data (Lipsey and Wilson, 2001). Further, in keeping with the best meta-analytic practices and the distortion effect large sample-size outliers may have on meta-analytic parameter estimates (see Fuller and Hester, 1999), a sensitivity analysis was conducted when large sample-size outliers were present in a group of studies. Similar to Balwant (2016), Z scores were calculated (i.e., “Critical Ratio Z” test; Hunter and Schmidt, 2004) to indicate the extent to which the estimated effect size estimates are different from effect size estimates of prior meta-analyses.

Finally, relative weight analysis was conducted using RWA-Web (Tonidandel and LeBreton, http://relativeimportance.davidson.edu) to assess the relative importance of the four NGL variables as predictors of the outcomes included in the aforementioned meta-analyses (Tonidandel and LeBreton, 2011). Relative weight analysis provides accurate estimates of the predictor variables' relative importance when those predictor variables are correlated, as they are in this case (Tonidandel and LeBreton, 2015, p. 208). Since the relative weights generated for each predictor are scaled in terms of variance explained, these values can, therefore, be interpreted as relative effect size estimates (Tonidandel and LeBreton, 2015). Consequently, these values are useful in making determinations of the practical utility of a variable or variables (Tonidandel and LeBreton, 2011).

For the sake of consistency, Hoch et al.'s (2018) results for most of the TL relationship data (i.e., engagement, job satisfaction, organizational commitment, trust in leader, LMX) were used, as it is the only study that analyzed all four NGL forms. However, because (a) research has shown that self-reports tend to exhibit larger effect sizes than other reports (e.g., Fuller et al., 1996; see also current results for EL-OCB) and (b) personal communication Hoch et al. (2018) combined self- and other-reports for their job performance and OCB analyses, Wang et al.'s (2011) results were used for job performance and OCB values as they separated out self- from other-reports. The correlation values reported in Tables 1–**4** were used as inputs to this analysis. The following were used as dependent variables in the relative weight analysis: job performance (other-reports), OCB (other-reports), CWB (self-reports), LMX, organizational commitment, job satisfaction, work engagement, intention to leave, and trust in supervisor. For the purposes of comparison with Hoch et al. (2018), a comparison was conducted of the *R*^2^ computed for the combined NGL forms (the combined model) with the *R*^2^ computed for TL.

## Results

Table 2 summarizes the meta-analytic results for EL. The estimated mean corrected correlation between EL and job performance (ρ = 0.31) was substantially stronger (i.e., a statistically significant difference) than reported in all three other meta-analytic studies (ρ = 0.25, ρ = 0.21, and ρ = 0.22 for Ng and Feldman, 2015; Bedi et al., 2016; Hoch et al., 2018, respectively). Similar results were obtained for the relationship between EL and OCB (*Z* = 2.48^*^; ρ = 0.37 self-report and ρ = 0.29 other-report). The results also indicate that self-reports of OCB exhibited a significantly stronger mean estimated effect size than other reports (*Z* = 2.26^*^), consistent with the common method bias perspective. The population estimate of CWB significantly differed from that of Hoch et al. (2018), yielding a population correlation estimate of −0.26/−0.25 compared with −0.45 (*Z* = 3.70^*^ and *Z* = 3.89^*^, respectively). LMX was found to have a significantly stronger population correlation estimate than that of Ng and Feldman (2015) with a *Z* score of 1.81^*^ (ρ = 0.78 and ρ = 0.60, respectively). Regarding the leadership styles, the results indicate the TL has a population correlation estimate of ρ = 0.83, which is significantly different from Hoch et al.'s (2018) estimate (ρ = 0.70; *Z* = 2.87^*^). EL was found to have population correlation estimates of 0.85 and 0.79 with AL and SL, respectively.

**Table 2:** Comparison of current and prior meta-analytic results for ethical leadership.

**Variable**	***K***	***N***	$\bar{r}$	**ρ**	***SD*** **ρ**	**CI_**LL**_ CI_**UL**_**	***Z***
**Job performance**							
Current study	29	7,175	0.27	0.31	0.16	(0.25 0.37)	
Hoch et al. (2018)^d^	22	4,904	0.22	0.25	0.06	(0.21 0.29)	1.85*
Ng and Feldman (2015)^a^	12	2,879	0.19	0.21	0.07	(0.17 0.25)	2.78*
Bedi et al. (2016)	16	3,741	0.22	0.22	0.10	(0.18 0.26)	2.32*
**Organizational citizenship behavior**							
Current study							
Self-report	26	6,460	0.31	0.37	0.14	(0.31 0.42)	
Other-report	29	7,246	0.25	0.29	0.12	(0.25 0.33)	2.26*
Hoch et al. (2018)^d^	22	5,049	0.25	0.29	0.08	(0.25 0.34)	2.48*/0.00
Ng and Feldman (2015)							
Self-report^a^	10	2,472	0.27	0.32	0.09	(0.26 0.38)	1.26
Other-report	16	3,530	0.21	0.24	0.09	(0.20 0.28)	1.59
Bedi et al. (2016)	17	3,958	0.37	0.39	0.27	(−0.15 0.92)	0.28/1.45
**Counterproductive work behavior**							
Current study	31	8,814	−0.23	−0.26	0.17	(−0.31 −0.20)	
Without sample-size outlier	30	7,289	−0.22	−0.25	0.17	(−0.30 −0.19)	0.23
Hoch et al. (2018)^d^	26	10,889	−0.39	−0.45	0.21	(−0.53 −0.38)	3.70*/3.89*
Ng and Feldman (2015)^a^	11	2,246	−0.31	−0.34	0.13	(−0.42 −0.26)	1.61/1.80*
Bedi et al. (2016)	8	1,807	−0.34	−0.36	0.51	(−0.41 −0.31)	0.39/0.60
**Job satisfaction**							
Current study	38	10,751	0.46	0.52	0.14	(0.48 0.57)	
Hoch et al. (2018)	17	4,578	0.45	0.50	0.11	(0.44 0.56)	0.51
Ng and Feldman (2015)^a^	10	2,983	0.37	0.42	0.21	(0.29 0.55)	1.42
Bedi et al. (2016)	18	5,744	0.56	0.64	0.30	(0.61 0.67)	1.62
**Organizational commitment**							
Current Study	56	13,737	0.39	0.45	0.19	(0.41 0.50)	
Without sample-size outlier	55	12,471	0.41	0.47	0.19	(0.42 0.52)	0.55
Hoch et al. (2018)	14	3,835	0.39	0.44	0.13	(0.36 0.51)	0.23/0.74
Ng and Feldman (2015)^a^	17	4,656	0.35	0.40	0.13	(0.34 0.46)	0.23/0.53
Bedi et al. (2016)	17	5,193	0.45	0.49	0.32	(0.46 0.52)	0.24/0.76
**Work engagement**							
Current study	17	4,673	0.42	0.46	0.16	(0.38 0.53)	
Hoch et al. (2018)	6	1,335	0.35	0.39	0.10	(0.29 0.48)	1.24
Ng and Feldman (2015)^a^	-	-	-	-	-	-	-
Bedi et al. (2016)	7	1,463	0.37	0.39	0.44	(0.33 0.44)	0.41
**Leader-member exchange**							
Current study	31	7,055	0.70	0.78	0.13	(0.74 0.82)	
Hoch et al. (2018)	18	4,052	0.65	0.71	0.20	(0.63 0.81)	1.33
Ng and Feldman (2015)^b^	11	3,184	0.54	0.60	0.32	(0.41 0.79)	1.81*
Bedi et al. (2016)	6	1,377	0.73	0.93	0.45	(0.87 0.98)	2.29*
**Trust in supervisor**							
Current Study	25	7,643	0.68	0.77	0.17	(0.70 0.84)	
Without sample-size outlier	24	6,383	0.68	0.76	0.19	(0.69 0.83)	0.19
Hoch et al. (2018)	18	4,105	0.58	0.66	0.27	(0.54 0.79)	1.52/1.33
Ng and Feldman (2015)	11	2,766	0.67	0.77	0.08	(0.72 0.82)	0.00/0.22
**Intention to leave**							
Current study	19	7,107	−0.35	−0.38	0.11	(−0.43 −0.33)	
Without sample-size outliers	17	5,075	−0.35	−0.39	0.13	(−0.45 −0.36)	0.25
Hoch et al. (2018)	7	2,942	−0.34	−0.37	0.11	(−0.46 −0.29)	0.21
Ng and Feldman (2015)	5	2,091	−0.35	−0.39	0.13	(−0.50 −0.28)	0.16
Bedi et al. (2016)	5	2,747	−40	−0.42	0.55	(−0.46 −0.38)	0.16
**Transformational leadership**							
Current Study	32	6,746	0.75	0.83	0.14	(0.78 0.87)	
Hoch et al. (2018)	20	3,717	0.63	0.70	0.17	(0.62 0.79)	2.87*
Ng and Feldman (2015)	13	2,426	0.69	0.76	0.17	(0.67 0.85)	1.31
Bedi et al. (2016)	5	856	0.94^c^	1.72^c^	0.49	(1.65 1.79)^c^	n.a.
**Authentic leadership**							
Current study	10	1,340	0.79	0.85	0.19	(0.73 0.97)	
Hoch et al. (2018)	-	-	-	-	-	-	-
Ng and Feldman (2015)	-	-	-	-	-	-	-
Bedi et al. (2016)	-	-	-	-	-	-	-
Lee et al. (2020)	3	462	0.77	0.85	0.15	(0.56 0.98)	0.00
**Servant leadership**							
Current Study	8	1,293	0.72	0.79	0.16	(0.68 0.89)	
Hoch et al. (2018)	-	-	-	-	-	-	-
Ng and Feldman (2015)	-	-	-	-	-	-	-
Bedi et al. (2016)	-	-	-	-	-	-	-
Lee et al. (2020)	4	3,106	0.74	0.82	0.11	(0.62 0.86)	0.38

K, number of studies; N, total sample size; r bar, average observed effect size; ρ, r bar corrected for measurement error in both the predictor and the criterion; SDρ, observed standard deviation in corrected correlations; CI_LL_ and CI_UL_, lower and upper bounds, respectively, of the 95% confidence interval around the mean true-score correlation; Z, Critical Ratio Z-score. A value of 1.64 or greater indicates a statistically significant difference (^*^p < 0.05) in mean true scores.

a *Includes both individual and group-level data*.

b *Includes experimental studies*.

c *d value(s)*.

d *Includes both self- and other-reports of outcome variable, Hoch et al. (2018) personal communication*.

Table 3 summarizes the meta-analytic results for SL. CWB was found to have a negative population correlation estimate of −0.39 with SL, which is larger than that of Lee et al. (2020; ρ = −0.27) but not significantly different (*Z* = 0.84). Regarding attitudinal measures, job satisfaction yielded a population correlation estimate that is significantly different from that of Hoch et al. (2018; *Z* = 2.67^*^; ρ = 0.52 and ρ = 0.66, respectively). LMX yielded a population correlation estimate much larger than that of Hoch et al. (2018) and Lee et al. (2020; *Z* = 2.19^*^; ρ = 0.81, ρ = 0.65, and ρ = 0.62, respectively). Regarding the leadership styles, the population correlation estimate associated with TL differed significantly from that reported by Hoch et al. (2018) and Lee et al. (2020; *Z* = 4.20^*^ and 5.68^*^; ρ = 0.77, ρ = 0.52, and ρ = 0.52, respectively). Lastly, the relationship between SL and AL was found to have a large positive population correlation estimate of 0.74, which is smaller, yet not significantly different from that of Lee et al. (2020; ρ = 0.84). Table 4 summarizes the meta-analytic results for AL. Job performance was more strongly related to AL (ρ = 0.23) than reported by Hoch et al. (2018; ρ = 0.12; *Z* = 2.03^*^). Moreover, CWB had a larger population correlation estimate than Hoch et al. (2018) with a value of −0.34 compared to −0.25 (*Z* = 1.70^*^). Lastly, the population correlation estimates for TL (ρ = 0.82) significantly differed from that of Banks et al. (2016; ρ = 0.72; *Z* = 1.70^*^). It is worthy to note that the number of studies and observations for the analyses relating TL to the other three leadership forms (i.e., TL-AL, TL-EL, and TL-SL) are substantially greater in the present study than in prior meta-analyses with an average number of studies and observations of 31 studies and 6,056 observations.

**Table 3:** Comparison of current and prior meta-analytic results for servant leadership.

**Variable**	***K***	***N***	$\bar{r}$	**ρ**	***SD*** **ρ**	**CI_**LL**_ CI_**UL**_**	***Z***
**Job performance**							
Current study	10	3,307	0.26	0.30	0.19	(0.18 0.42)	
Hoch et al. (2018)^a^	8	2,077	0.20	0.23	0.08	(0.15 0.31)	1.05
Lee et al. (2020)	26	7,711	0.23	0.25	0.13	(0.18 0.27)	0.77
**Organizational citizenship behavior**							
Current study							
Self-report	8	3,491	0.33	0.40	0.12	(0.31 0.48)	
Other-report	20	4,920	0.31	0.35	0.16	(0.28 0.41)	0.90
Hoch et al. (2018)^a^	6	2,404	0.33	0.40	0.12	(0.28 0.51)	0.00/0.82
Lee et al. (2020)	40	13,418	0.34	0.39	0.18	(0.29 0.39)	0.20/0.87
**Counterproductive work behavior**							
Current study	6	1,868	−0.36	−0.39	0.29	(−0.63 −0.16)	
Hoch et al. (2018)	-	-	-	-	-	-	-
Lee et al. (2020)	9	4,186	−0.22	−0.27	0.24	(−0.36 −0.07)	0.84
**Job satisfaction**							
Current study	38	11,655	0.47	0.52	0.25	(0.44 0.60)	
Without sample-size outliers	36	8,697	0.51	0.57	0.27	(0.48 0.65)	0.83
Hoch et al. (2018)	11	2,671	0.60	0.66	0.11	(0.59 0.73)	2.67*/1.61
**Organizational commitment**							
Current study	30	9,661	0.55	0.62	0.26	(0.53 0.70)	
Without sample-size outlier	29	7,948	0.54	0.62	0.29	(0.51 0.72)	0.00
Hoch et al. (2018)	11	2,424	0.49	0.55	0.35	(0.33 0.76)	0.60/0.59
**Work engagement**							
Current study	13	3,846	0.46	0.50	0.22	(0.38 0.62)	
Hoch et al. (2018)	4	959	0.47	0.52	0.00	(0.47 0.58)	0.33
**Leader-member exchange**							
Current study	17	3,904	0.73	0.81	0.14	(0.74 0.88)	
Hoch et al. (2018)	4	938	0.59	0.65	0.16	(0.48 0.83)	2.19*
Lee et al. (2020)	14	4,171	0.52	0.62	0.20	(0.41 0.63)	3.00*
**Trust in supervisor**
Current study	19	4,717	0.66	0.75	0.14	(0.68 0.81)	
Hoch et al. (2018)	7	1,886	0.63	0.71	0.12	(0.58 0.82)	0.72
Lee et al. (2020)	12	2,884	0.57	0.67	0.14	(0.49 0.65)	1.55
**Intention to leave**
Current study	8	3222	−0.29	−0.34	0.01	(−0.38 −0.29)	
Without sample-size outlier	7	2261	−0.30	−0.35	0.01	(−0.40 −0.31)	1.93*
**Transformational leadership**							
Current study	27	4,579	0.69	0.77	0.17	(0.71 0.83)	
Hoch et al. (2018)	5	774	0.47	0.52	0.08	(0.45 0.60)	4.20*
Lee et al. (2020)	14	3,867	0.45	0.52	0.11	(0.40 0.51)	5.68*
**Authentic leadership**							
Current study	6	2,256	0.67	0.74	0.18	(0.59 0.88)	
Hoch et al. (2018)	-	-	-	-	-	-	-
Lee et al. (2020)	5	2,686	0.78	0.84	0.11	(0.67 0.89)	1.13
**Ethical leadership**
Current study	8	1,293	0.72	0.79	0.16	(0.68 0.89)	
Hoch et al. (2018)	-	-	-	-	-	-	-
Lee et al. (2020)	4	3,106	0.74	0.82	0.11	(0.62 0.86)	0.38

K, number of studies; N, total sample size; r bar, average observed effect size; ρ, r bar corrected for measurement error in both the predictor and the criterion; SDρ, observed standard deviation in corrected correlations; CI_LL_ and CI_UL_, lower and upper bounds, respectively, of the 95% confidence interval around the mean true-score correlation; Z, Critical Ratio Z-score. A value of 1.64 or greater indicates a statistically significant difference (*p < 0.05) in mean true scores.

a *Includes both self- and other-reports of outcome variable, Hoch et al. (2018) personal communication*.

**Table 4:** Comparison of current and prior meta-analytic results for authentic leadership.

**Variable**	***K***	***N***	$\bar{r}$	**ρ**	***SD*** **ρ**	**CI_**LL**_ CI_**UL**_**	***Z***
**Job performance**							
Current study	20	4,933	0.21	0.23	0.15	(0.17 0.30)	
Hoch et al. (2018)^a^	8	2,101	0.11	0.12	0.09	(0.04 0.20)	2.03*
Banks et al. (2016)	9	2,054	0.12	0.14	0.04	(0.08 0.19)	1.56
**Organizational citizenship behavior**							
Current study							
Self-report	22	5,160	0.32	0.40	0.20	(0.32 0.48)	
Other-report	13	2,281	0.28	0.33	0.24	(0.20 0.46)	0.89
Hoch et al. (2018)^a^	8	1,256	0.29	0.33	0.19	(0.19 0.47)	0.88/0.00
Banks et al. (2016)	10	2,309	0.42	0.48	0.24	(0.33 0.63)	0.92/1.49
**Counterproductive work behavior**							
Current study	10	3,450	−0.30	−0.34	0.11	(−0.40 −0.27)	
Hoch et al. (2018)	4	1,175	−0.22	−0.25	0.08	(−0.35 −0.14)	1.70*
Banks et al. (2016)	3	1,549	−0.28	−0.31	0.12	(−0.46 −0.17)	0.39
**Job satisfaction**
Current study	37	8,667	0.44	0.50	0.17	(0.45 0.55)	
Hoch et al. (2018)	9	2,129	0.44	0.48	0.09	(0.42 0.55)	0.49
Banks et al. (2016)	16	4,084	0.48	0.53	0.16	(0.45 0.61)	0.61
**Organizational commitment**							
Current study	32	7,442	0.44	0.52	0.17	(0.46 0.57)	
Hoch et al. (2018)	5	797	0.43	0.48	0.12	(0.36 0.61)	0.65
Banks et al. (2016)	17	4,077	0.44	0.51	0.16	(0.43 0.59)	0.20
**Work engagement**							
Current study	37	10,662	0.38	0.42	0.24	(0.35 0.50)	
Without effect-size outlier	36	10,336	0.42	0.46	0.15	(0.41 0.50)	0.86
Hoch et al. (2018)	6	1,182	0.43	0.47	0.18	(0.30 0.62)	0.64/0.13
Banks et al. (2016)	11	3,018	0.33	0.37	0.38	(0.14 0.59)	0.41/0.77
**Leader-member exchange**							
Current study	16	5,142	0.69	0.75	0.10	(0.70 0.79)	
Hoch et al. (2018)	4	1,468	0.62	0.67	0.17	(0.47 0.85)	0.90
Banks et al. (2016)	6	2,083	0.60	0.65	0.22	(0.47 0.83)	1.07
**Trust in supervisor**							
Current study	22	5,619	0.66	0.74	0.20	(0.65 0.82)	
Hoch et al. (2018)	6	929	0.64	0.69	0.18	(0.54 0.84)	0.59
Banks et al. (2016)	12	3,210	0.57	0.65	0.19	(0.54 0.76)	1.30
**Intention to leave**							
Current study	9	3,425	−0.29	−0.33	0.08	(−0.38 −0.28)	
Banks et al. (2016)	5	1,149	−0.20	−0.21	0.31	(−0.49 0.06)	0.85
**Transformational leadership**							
Current study	34	6,842	0.73	0.82	0.10	(0.78 0.85)	
Hoch et al. (2018)	10	2,397	0.67	0.75	0.26	(0.58 0.92)	0.83
Banks et al. (2016)	23	5,414	0.70	0.72	0.27	(0.60 0.83)	1.70*
**Ethical leadership**							
Current Study	10	1,340	0.79	0.85	0.19	(0.73 0.97)	
Hoch et al. (2018)	-	-	-	-	-	-	-
Lee et al. (2020)	3	462	0.77	0.85	0.15	(0.56 0.98)	0.00
**Servant leadership**							
Current study	6	2,256	0.67	0.74	0.18	(0.59 0.88)	
Hoch et al. (2018)	-	-	-	-	-	-	-
Lee et al. (2020)	5	2,686	0.78	0.84	0.11	(0.67 0.89)	1.13

K, number of studies; N, total sample size; r bar, average observed effect size; ρ, r bar corrected for measurement error in both the predictor and the criterion; SDρ, observed standard deviation in corrected correlations; CI_LL_ and CI_UL_, lower and upper bounds, respectively, of the 95% confidence interval around the mean true-score correlation; Z, Critical Ratio Z-score. A value of 1.64 or greater indicates a statistically significant difference (*p < 0.05) in true mean scores. Mean true scores.

a *Includes both self- and other-reports, Hoch et al. (2018) personal communication*.

Table 5 displays a summary of the results of the current effort and prior meta-analyses that investigated the relationship between all the variables compared in this study. Along with the new estimates for TL, the average mean corrected correlation estimate across the four NGL constructs was very high (0.80). This average is well above the level at which most scholars conclude there is a lack of discriminant validity. LMX is also shown to be highly correlated with the four NGL (ranging from ρ = 0.71 to ρ = 0.81). Overall, the most notable differences from prior studies are in the relationship estimates for the leadership variables (LMX, EL, AL, SL, and TL), with the current results being stronger in magnitude than estimated in prior studies. Table 5 also shows that the four NGL tend to have similar magnitude effect sizes, with the exception of organizational commitment and CWB.

**Table 5:** Meta-analytic correlations summary from current and prior studies.

**Variable**	**1**	**2**	**3**	**4**
1. Transformational leadership	1			
2. Authentic leadership	0.82	1		
3. Ethical leadership	0.83	0.85/0.85^f^	1	
4. Servant leadership	0.77/0.52^f^	0.74/0.84^f^	0.79/0.82^f^	1
5. Leader-member exchange	0.71^c^/0.73^d^	0.75	0.78	0.81/0.62^f^
6. Engagement	0.48^c^	0.42	0.46	0.50
7. Job satisfaction	0.42^c^	0.50	0.52	0.52
8. Org. commitment	0.43^c^	0.52	0.45	0.62
9. Job performance^a^	0.25^e^	0.23	0.31	0.30/0.25^f^
10. Counterproductive behavior^b^	−0.23^c^	−0.34	−0.26	−0.39/−0.27^f^
11. Organizational citizenship^a^	0.30^e^	0.33	0.29	0.35/0.39^f^
12. Trust in supervisor	0.65^c^	0.74	0.77	0.75/0.67^f^
13. Intention to leave	−0.31	−0.33	−0.38	−0.34

*Meta-analytic correlations without a note were calculated in this study*.

a *Other-report*.

b *Self-report*.

c *Hoch et al. (2018)*.

d *Dulebohn et al. (2012)*.

e *Wang et al. (2011)*.

f *Lee et al. (2020)*.

The results of the relative weight analysis are presented in Table 6. The values reported in Table 6 are estimates of the percentage (i.e., proportion) of total predicted variance in the outcome variable accounted for by each leadership variable (i.e., rescaled relative weights; Tonidandel and LeBreton, 2015). Note that because the sample-size outlier sensitivity analyses showed either little or no difference in effect size estimates when the outlier(s) (samples > 1,000 observations) were removed, the information generated from the full samples was used for the relative weight analyses. The results indicate that each leadership form accounts for some degree of unique variance in each outcome variable. Regarding relative importance, SL accounted for the largest proportion of total variance in 6 of 9 models (CWB, LMX, organizational commitment, job satisfaction, work engagement, and trust in supervisor), followed by EL accounting for the largest proportion of total predicted variance in the remaining three models (OCB, job performance, and intention to leave). In comparison, TL accounted for the smallest proportion of total variance in 7 of 9 models, and AL accounted for the smallest proportion of total variance in 2 of 9 models. In terms of the average proportion of total variance accounted for across the nine models, SL accounted for an average of 34.4% of the total variance, followed by EL (25.9%), AL (22.0%), and TL (17.7%).

**Table 6:** Relative weight analysis for transformational, authentic, ethical, and servant leadership.

**Leadership variable**	**OCB**	**Job performance**	**CWB**	**LMX**	**Organizational commitment**	**Job satisfaction**	**Work engagement**	**Intention to leave**	**Trust in supervisor**
Transformational	15.49	20.33	11.86	18.74	13.02	14.01	27.14	16.83	22.15
Authentic	21.79	13.47	29.03	22.81	24.41	25.62	16.32	21.23	23.76
Ethical	32.96	34.45	13.57	25.03	14.46	28.05	21.75	36.10	26.37
Servant	29.75	31.74	45.53	34.08	48.11	32.31	34.79	25.84	27.72
*R*^2^ of combined forms	0.15	0.11	0.21	0.72	0.43	0.32	0.27	0.15	0.66
*R*^2^ of transformational	0.09	0.07	0.05	0.50	0.19	0.18	0.23	0.10	0.42
*% Increase in R*^2^ of combined forms over transformational	55%	57%	400%	44%	221%	78%	17%	50%	57%
*R*^2^ of servant	0.12	0.09	0.15	0.66	0.38	0.27	0.25	0.12	0.56
*% Increase in R*^2^ of combined forms over servant	25%	22%	40%	9%	13%	19%	8%	25%	18%

*Organizational citizenship behavior and job performance are other-report data (i.e., multi-source); counterproductive workplace is self-report data; OCB, organizational citizenship behavior; CWB, counterproductive workplace behavior; LMX, leader-member exchange; Combined Forms, all four leadership forms are entered into the model*.

The assessment of the incremental variance in outcomes gained by AL, EL, and SL over TL indicates that the addition of the three newer forms of leadership account for an average of 108.7% additional variance in the outcome variables (i.e., the summation of % increase in *R*^2^ of combined forms over transformational from OCB to trust in supervisor divided by 9, see Table 6). Because the results of the relative weight analysis mostly indicate that SL is the most important leadership form relative to the other three leadership forms, a similar analysis was conducted to assess the extent to which TL, AL, and EL added incremental variance beyond that accounted for by SL. The average incremental variance in the outcomes accounted for by adding TL, AL, and EL to SL is still a substantial 20% (from OCB to trust in supervisor, see Table 6).

## Discussion

This study provides a meta-analytic assessment of the three moral/value-based leadership styles (i.e., EL, SL, and AL; Lemoine et al., 2019). For the variables common to the analyses and Hoch et al. (2018), substantially more studies and observations in the analyses were aggregated in this study compared to Hoch et al. (2018; average of 28 vs. 11 studies; average of 7,107 vs. 2,660 observations). Consequently, this study answers the call of scholars (i.e., Hoch et al., 2018; Eva et al., 2019) for future meta-analyses of NGL forms to aggregate more studies of prior research so there can be greater confidence in the stability of the results and the conclusions drawn from those results. The results indicate that the relationship between TL and the other leadership forms is even stronger than reported in previous meta-analyses. The magnitude of the increase in mean estimated effect size for the relationship between TL and SL is one of the most noteworthy findings (Hoch et al., 2018, ρ = 0.52; Lee et al., 2020, ρ = 0.52; current study, ρ = 0.77), strongly suggesting empirical redundancy—which is different from Hoch et al.'s (2018) conclusion that the two are empirically distinct. Although the average mean corrected correlation between SL and the other leadership forms was found to be somewhat less than the average estimated correlations among the other three leadership forms (ρ = 0.76 vs. ρ = 0.83), the magnitude of the SL relationships is still high enough to be suggestive of redundancy. Further, the results indicate there are very strong intercorrelations between AL, EL, and SL, with an average mean corrected correlation of 0.79. Accordingly, the results provide strong meta-analytic evidence indicating that all four of the NGL forms share a large common core and questionable distinctiveness.

There were relatively few *Z* scores indicating significant differences across the different meta-analytic studies (18 out of 82 comparisons—differences in roughly 22% of comparisons). Even so, some differences from prior research are noteworthy, such as the increase in effect size estimates of the job performance and LMX relationships. In contrast to the conclusions reached by Banks et al. (2018) and Hoch et al. (2018), the results indicate TL does not have the strongest relationship with job performance—both EL and SL have stronger relationships with job performance than TL. This result is corroborated by the relative weight analysis. The results also provide stronger indications that LMX may be redundant with AL, EL, and SL than reported in prior meta-analyses.

Furthermore, building upon prior meta-analyses that used simple paired comparisons of the relative weights of TL to AL, EL, and SL (e.g., Hoch et al., 2018), this study provides more insight into the relative importance of all four leadership forms. More specifically, because the omitted-variable-based endogeneity bias present in prior meta-analytic research was accounted for in this study, a better understanding of the effects of each of the four leadership forms was reached across a variety of behavioral, relational, and follower-oriented variables even beyond those tested in prior meta-analyses (e.g., OCB, job performance, and CWB; Lee et al., 2020). While Hoch et al. (2018) found AL, EL, and SL to have only marginally larger average relative weights than TL, the findings of this study show that the relative weights for AL, EL, and SL are substantially larger than predicted for TL. Indeed, the average percentage of total predicted variance across all 9 outcome variables for SL was approximately twice as large as that for TL. Overall, SL was found to have the highest average relative importance, followed by EL, AL, and TL. This clear pattern of relative importance did not vary substantially across behavioral, relational, or follower-oriented outcomes groups. This new finding is noteworthy because it indicates that even though the four leadership forms are very strongly correlated, there is a substantial deficiency in the predictive validity of TL that can be substantially reduced by including AL, EL, and SL, indicating the incremental utility of the three moral/value-based leadership forms above and beyond TL.

Moreover, AL, EL, and SL accounted for a very large average increase in *R*^2^ over that accounted for by TL alone. These results add additional weight to Hoch et al. (2018)'s conclusion that TL has some deficit in ethical/moral content relative to the other three leadership forms. The average increase in *R*^2^ provided by TL, AL, and EL over SL alone was substantially less than found in the TL alone analysis, although it is still large enough that it is likely to be considered practically significant. That is, even the most relatively important leadership form (i.e., SL) benefits substantially from adding the other three leadership forms to predictive models. Furthermore, the findings of the relative weight analysis differ from those of Lee et al. (2020) in terms of the relative importance of each leadership style in predicting OCB, job performance, and CWB. Whereas, Lee et al. (2020) found EL to account for the least relative contribution to predicting OCB and job performance, the findings of this study indicate that it is the most important contributor. On the other hand, whereas Lee et al. (2020) concluded that EL accounts for the most relative contribution to predicting CWB, the findings of this study indicate that it is the third most contributor. Given the larger number of studies to draw conclusion from, including the intercorrelations between the leadership forms, this study suggests that organizations would be better off training leaders in ethical leadership than servant leadership to improve an individual's OCB and job performance and minimize their intention to leave.

Based upon these results, the question becomes, “where do leadership scholars go from here?” One way forward for leadership researchers and the field of leadership involves integrating what researchers know about the four new genre leadership forms rather than continuing to seek a way to better differentiate the individual theories and measures. The principle of parsimony would suggest that a Grand Unified Approach would represent a useful step forward over continuing pursuing separate research agendas for each individual leadership form (Banks et al., 2018). Pursuing this path to greater parsimony could refocus the efforts on leadership scholars such that the insights gained from future research are not diffused by pursuing further research on individual leadership forms (“disjunctivitis;” Antonakis, 2017). The finding provides compelling enough evidence for future research to answer Banks et al. (2018) call for a measure integrating the four forms of leadership. One possible description for such integrative measure can be represented in the potential construct of *Value Convergent Leadership*, to capture the idea that all four leadership forms have, at least to some degree, a values orientation (Hoch et al., 2018) and operate on subordinate perceptions that their own values converge with the values espoused or exhibited by the leader whether related to the vision, decision-making process, or leader behavior (Lemoine et al., 2019). Creating this new measure enables leadership scholars to provide some preliminary answers to questions posed by Banks et al. (2018), including how well would this omnibus construct be related to outcomes such as workplace deviance, empowerment, LMX, and trust in leader?

## Limitations and Future Directions

Like any research, this research has limitations. Although some scholars include spiritual leadership in the same category as EL, SL, and AL (e.g., Dinh et al., 2014), it was not included in the meta-analytic examination of the literature due to the lack of sufficient studies. Second, due to space constraints, no analyses at the group level were included. Therefore, suggestions that some leadership forms may be more effective at the group level than at the individual level could not be addressed in this study (Banks et al., 2018).

Future research should focus on creating an integrative measure of the NGL forms and assess its overlap with other leadership constructs, such as spiritual, paradoxical, or empowering leadership. As such measure ought to be a *compound* leadership construct, it may be that spiritual leadership could also be integrated into the grand unified approach conceptually and empirically to gain further parsimony in the field. Although this pathway forward requires more construct validity research, it has the potential for greater progress than a more fragmented approach represented by the separate NGL forms.

## Data Availability Statement

The original contributions presented in the study are included in the article/Supplementary Material, further inquiries can be directed to the corresponding author.

## Author Contributions

BF: conceptualization, wrote–original draft, methodology, and formal analysis. AB: investigation, wrote–review and editing, data collection, and project administration. SB: funding acquisition, resources, wrote–review and editing, and data collection. All authors contributed to the article and approved the submitted version.

## Conflict of Interest

The authors declare that the research was conducted in the absence of any commercial or financial relationships that could be construed as a potential conflict of interest.

## Publisher's Note

All claims expressed in this article are solely those of the authors and do not necessarily represent those of their affiliated organizations, or those of the publisher, the editors and the reviewers. Any product that may be evaluated in this article, or claim that may be made by its manufacturer, is not guaranteed or endorsed by the publisher.
