# Supplementary material for: Enhancing and Extending the Meta-Analytic Comparison of Newer Genre Leadership Forms
Source: Front Psychol. 2022 Apr 12;13:872568. doi: 10.3389/fpsyg.2022.872568 (PMC9039396; doi:10.3389/fpsyg.2022.872568)
Supplement: Supplementary file 1 [file Table_1.DOCX]

**SUPPLEMENTARY APPENDIX**

**STUDIES INCLUDED IN THE META-ANALYSIS**

Agote, E. L. (2013). Authentic leadership, trust & followers’ emotions: The experience of HRMs during organizational change processes. *Azkoaga*, 16, 123-148.

Ahmad, I., & Gao, Y. (2017). Ethical leadership & follower’s work attitude: The role of moral identification. *Research on Humanities & Social Sciences*, 7(18), 124-130.

Ahn, J., Lee, S., & Yun, S. (2018). Leaders’ core self-evaluation, ethical leadership, & employees’ job performance: The moderating role of employees’ exchange ideology. *Journal of Business Ethics*, 148(2), 457-470.

Akker, L. V., Heres, L., Lasthuizen, K., & Six, F. E. (2009). Ethical leadership & trust: It's all about meeting expectations. *International Journal of Leadership Studies,* 5(2), 102-122.

Al-Yousef, B. (2012). *Servant leadership perception & job satisfaction among SFDA employees in Saudi Arabia—A correlational study*.

Ali, N., Jan, S., Ali, A., & Tariq, M. (2014). Transformational & transactional leadership as predictors of job satisfaction, commitment, perceived performance & turnover intention (empirical evidence from Malak& division, Pakistan). *Life Science Journal*, 11(5), 48-53.

Alzoman, M. (2012). *Effective leadership of a culturally diverse workforce in Saudi Arabia Basic Industries Corporation (SABIC)*. Unpublished doctoral dissertation, University of Portsmouth.

Amadeo, C. A. (2008). *A correlational study of servant leadership & registered nurse job satisfaction in acute health-care settings*. Unpublished doctoral dissertation, University of Phoenix.

Amah, O. E. (2018). Determining the antecedents & outcomes of servant leadership. *Journal of General Management*, 43(3), 126–138.

&erson, T. L. K. (2011). *Relationships between chief nurse executive perceived leadership styles, personal attributes & organizational context*. Unpublished doctoral dissertation, College of Saint Mary.

Arain, G. A. (2018) The Impact of Leadership Style on Moral Identity & Subsequent In-Role Performance: A Moderated Mediation Analysis. *Ethics & Behavior*, 28(8), 613-627.

Ariyabuddhiphongs, V., & Kahn, S. I. (2017). Transformational leadership & turnover intention: The mediating effects of trust & job performance on café employees in Thail&. *Journal of Human Resources in Hospitality & Tourism*, 16(2), 215-233.

Asag-Gau, L., & Van Dierendonck, D. (2011). The impact of servant leadership on organisational commitment among the highly talented: the role of challenging work conditions & psychological empowerment. *European Journal of International Management*, 5(5), 463-483.

Avey, J. B., Palanski, M. E., & Walumbwa, F. O. (2011). When leadership goes unnoticed: The moderating role of follower self-esteem on the relationship between ethical leadership & follower behavior. *Journal of Business Ethics*, 98, 573-582.

Avey, J. B., Wernsing, T. S., & Palanski, M. E. (2012). Exploring the process of ethical leadership: The mediating role of employee voice and psychological ownership. *Journal of Business Ethics*, 107, 21-34.

Awee, A., Cheah, W. Y., Cheng, C. N., Hong, K. S., Ling, L. B., & Tan, M. C. (2014). Effect of servant leadership on affective commitment among hotel employees. *International Journal of Scientific and Research Publications*, 4(11), 1-7.

Azanza, G., Moriano, J. A., Molero, F., & Lévy Mangin, J. P. (2015). The effects of authentic leadership on turnover intention. *Leadership & Organization Development Journal*, 36(8), 955-971.

Babakus, E., Yavas, U., & Ashill, N. J. (2010). Service worker burnout and turnover intentions: Roles of person-job fit, servant leadership, and customer orientation. *Services Marketing Quarterly*, 32(1), 17-31.

Babalola, M. T., Stouten, J., & Euwema, M. (2016). Frequent Change and Turnover Intention: The Moderating Role of Ethical Leadership. *Journal of Business Ethics,* 134(2), 311-322.

Babalola, M. T., Stouten, J., Camps, J., & Euwema, M. (2019). When do ethical leaders become less effective? The moderating role of perceived leader ethical conviction on employee discretionary reactions to ethical leadership. *Journal of Business Ethics*, 154, 85-102.

Badrinarayanan, V., Ramachandran, I., & Madhavaram, S. (2019). Mirroring the boss: Ethical leadership, emulation intentions, and salesperson performance. *Journal of Business Ethics*, 159(3), 897-912.

Banks, G. C., Mccauley, K. D., Gardner, W. L., & Guler, C. E. (2016). A meta-analytic review of authentic and transformational leadership: A test for redundancy. *The Leadership Quarterly,* 27(4), 634-652.

Barbuto Jr., J. E., & Wheeler, D. W. (2006). Scale development and construct clarification of servant leadership. *Group and Organization Management*, 31(3), 300-326.

Barbuto Jr., J. E., Singh, M., Wilmot, M. P., & Story, J. S. (2012). Self-other rating agreement and leader-member exchange (LMX): A quasi-replication. *Perceptual and motor skills*, *114*(2), 479-484.

Basar, U., & Filizoz, B. (2015). Can ethical leaders heal the wounds? An empirical research. *Eurasian Journal of Business and Economics*, 8(15), 199-218.

Becerra, X. H. (2016). *The effects of pastoral servant leadership and commitment of members to the organization in Latin American and Anglo American congregations: As mediated by leader-member exchange and identification with the leader.* Unpublished doctoral dissertation, Regent University.

Bedi, A., Alpaslan, C., & Green, S. (2016). A Meta-analytic Review of Ethical Leadership Outcomes and Moderators. *Journal of Business Ethics,* 139(3), 517-536.

Beeri, I., Dayan, R., Vigoda-Gadot, E., & Werner, S. B. (2013). Advancing ethics in public organizations: The impact of an ethics program on employees’ perceptions and behaviors in a regional council. *Journal of Business Ethics,* 112*,* 59-78.

Benevene, P., Dal Corso, L., De Carlo, A., Falco, A., Carluccio, F., & Vecina, M. L. (2018). Ethical leadership as antecedent of job satisfaction, affective organizational commitment and intention to stay among volunteers of non-profit Organizations. *Frontiers in Psychology*, 9.

Biyik, Y, Simsek, T, Erden, P. (2017). Efficiency of ethical leadership workers to business performance and business satisfaction. *Gazi Economics and Business Administration*, 3(1), 59-70.

Bobbio, A., Dierendonck, D. V., & Manganelli, A. M. (2012). Servant leadership in Italy and its relation to organizational variables. *Leadership*, 8(3), 229-243.

Bonner, J., Greenbaum, R., & Mayer, D. (2016). My Boss is Morally Disengaged: The Role of Ethical Leadership in Explaining the Interactive Effect of Supervisor and Employee Moral Disengagement on Employee Behaviors. *Journal of Business Ethics,* 137(4), 731-742.

Borchert, D. (2011). *A meta-model of ethical behavior: An empirical examination of ethical leadership, ethical identity, ethical climate, and emotions on unethical work behavior*. Unpublished doctoral dissertation, Saint Louis University.

Borgmann, L., & Rowold, J. The relationship between leadership styles and relevant organizational outcome criteria in a department store chain*.* *Unpublished manuscript*.

Borgmann, L., & Rowold, J. Different Leadership styles and outcome criteria-an investigation in a police administration. *Unpublished article*.

Borgmann, L., & Rowold, J. On the relationship between leadership styles and relevant organisational outcome criteria in a German bank. *Unpublished article*.

Borgmann, L., & Rowold, J. On the relationship between leadership styles and relevant organisational outcome criteria in a German insurance company. *Unpublished article*.

Borgmann, L., & Rowold, J. On the relationship between leadership styles and relevant organisational outcome criteria in the public sector. *Unpublished article*.

Borgmann, L., & Rowold, J. The relationship between leadership styles and relevant organisational outcome criteria in a snowball sample. *Unpublished article*.

Bormann, K. C. (2013). *Understanding ethical leadership: An integrative model of its antecedents, correlates, contingencies, and outcomes.* Unpublished doctoral dissertation, TU Dortmund University.

Bouckenooghe, D., Zafar, A., & Raja, U. (2015). How Ethical Leadership Shapes Employees' Job Performance: The Mediating Roles of Goal Congruence and Psychological Capital. *Journal of Business Ethics,* 129(2), 251-264.

Braun, S., & Nieberle, K. W. (2017). Authentic leadership extends beyond work: A multilevel model of work-family conflict and enrichment. *The Leadership Quarterly*, 28(6), 780-797.

Brennan, P. T. (2010). *Pursuing success without scandal: Exploring the relationship between transformational and authentic leadership*. Unpublished doctoral dissertation, Capella University, Minneapolis, MN.

Brown, A. B. (2014). *Teacher perceptions of the servant leadership characteristics of one principal in relation to job satisfaction*. Unpublished doctoral dissertation, Grand Canyon University.

Burch, T. C., & Guarana, C. L. (2014). The comparative influences of transformational leadership and leader–member exchange on follower engagement. *Journal of Leadership Studies*, 8(3), 6-25.

Burton, L. J., Welty Peachey, J., & Wells, J. E. (2017). The role of servant leadership in developing an ethical climate in sport organizations. *Journal of Sport Management*, 31(3), 229-240.

Butarbutar, I. D., Sendjaya, S., & Härtel, C. E. (2010). The mediating effects of ethical climate on the relationship between servant leadership and organizational citizenship behaviour. In *24th ANZAM Conference* (pp. 1-22).

Byun, G., Karau, S. J., Dai, Y., & Lee, S. (2018). A three-level examination of the cascading effects of ethical leadership on employee outcomes: A moderated mediation analysis. *Journal of Business Research*, 88, 44-53.

Caffey, R. D. (2012). *The relationship between servant leadership of principals and beginning teacher job satisfaction & intent to stay*. Unpublished doctoral dissertation, University of Missouri.

Caillier, J. G. (2016). Do transformational leaders affect turnover intentions and extra-role behaviors through mission valence? *The American Review of Public Administration*, 46(2), 226-242.

Caillier, J. G. (2016). Linking transformational leadership to self-efficacy, extra-role behaviors, and turnover intentions in public agencies: The mediating role of goal clarity. *Administration and Society*, 48(7), 883-906.

Cameron, R. K. (2007). *Governing ourselves before governing others: An investigation of authentic leadership*. Unpublished doctoral dissertation, Capella University.

Chan, S. C. (2014). Paternalistic leadership and employee voice: Does information sharing matter?. *Human Relations*, 67(6), 667-693.

Chan, S., & Mak, W. M. (2014). The impact of servant leadership and subordinates' organizational tenure on trust in leader and attitudes. *Personnel Review*, 43(2), 272-287.

Chen, A. S. Y., & Hou, Y. H. (2016). The effects of ethical leadership, voice behavior and climates for innovation on creativity: A moderated mediation examination. *The Leadership Quarterly,* 27(1), 1-13.

Chen, X. P., Eberly, M. B., Chiang, T. J., Farh, J. L., & Cheng, B. S. (2014). Affective trust in Chinese leaders: Linking paternalistic leadership to employee performance. *Journal of management*, 40(3), 796-819.

Chen, Z., Zhu, J., & Zhou, M. (2015). How Does a Servant Leader Fuel the Service Fire? A Multilevel Model of Servant Leadership, Individual Self Identity, Group Competition Climate, and Customer Service Performance. *Journal of Applied Psychology,* 100(2), 511-521.

Cheng, J., Chang, S. Kuo, J., & Cheung, Y. (2014). Ethical leadership, work engagement, and voice behavior. *Industrial Management and Data Systems,* 114, 817-831.

Chin, T. (2013). How ethical leadership encourages employee voice behavior in China: The mediating role of organizational harmony. *International Business Research*, 6*,* 15-24.

Chiniara, M., & Bentein, K. (2016). Linking servant leadership to individual performance: Differentiating the mediating role of autonomy, competence and relatedness need satisfaction. *The Leadership Quarterly,* 27(1), 124-141.

Chiniara, M., & Bentein, K. (2018). The servant leadership advantage: When perceiving low differentiation in leader-member relationship quality influences team cohesion, team task performance and service OCB. *The Leadership Quarterly*, 29(2), 333-345.

Chinomona, R., Mashiloane, M., & Pooe, D. (2013). The influence of servant leadership on employee trust in a leader and commitment to the organization. *Mediterranean Journal of Social Sciences*, 4(14), 405.

Cho, K. S., & Park, H. Y. (2015). Why Does Trust Mediate the Effects of Ethical and Authentic Leadership in Korean Firms? *Asian International Studies Review*, 16(2), 49-70.

Choudhary, A. I., Akhtar, S. A., & Zaheer, A. (2013). Impact of transformational and servant leadership on organizational performance: A comparative analysis. *Journal of Business Ethics*, 116(2), 433-440.

Chughtai, A. (2016). Servant Leadership and Follower Outcomes: Mediating Effects of Organizational Identification and Psychological Safety. *The Journal of Psychology,* 150(7), 866-880.

Chughtai, A., Byrne, M., & Flood, B. (2015). Linking Ethical Leadership to Employee Well-Being: The Role of Trust in Supervisor. *Journal of Business Ethics,* 128(3), 653-663.

Copeland, M. (2016). The Impact of Authentic, Ethical, Transformational Leadership on Leader Effectiveness. *Journal of Leadership, Accountability and Ethics,* 13(3), 79-97.

Copeland, M. K. (2013). *The importance of ethical leadership in the accounting industry.* Unpublished doctoral dissertation, Regent University.

Daams, R. J. C. (2012). *Ethical leadership and followers’ organizational compliance: Mediating role of psychological contract and moderating role of organizational identification.* Unpublished Master’s thesis, Tilburg University.

De Clercq, D., Bouckenooghe, D., Raja, U., & Matsyborska, G. (2014). Servant leadership and work engagement: The contingency effects of leader–follower social capital. *Human Resource Development Quarterly*, 25(2), 183-212.

Deconinck, J. (2015). Outcomes of ethical leadership among salespeople. *Journal of Business Research,* 68(5), 1086-1093.

Demirtas, O. (2015). Ethical Leadership Influence at Organizations: Evidence from the Field. *Journal of Business Ethics,* 126(2), 273-284.

Demirtas, O., & Akdogan, A. (2015). The Effect of Ethical Leadership Behavior on Ethical Climate, Turnover Intention, and Affective Commitment. *Journal of Business Ethics,* 130(1), 59-67.

Demirtas, O., Hannah, S. T., Gok, K., Arslan, A., & Capar, N. (2017). The moderated influence of ethical leadership, via meaningful work, on followers’ engagement, organizational identification, and envy. *Journal of Business Ethics*, 145(1), 183-199.

Den Hartog, D. N., & Belschak, F. D. (2012). Work engagement and Machiavellianism in the ethical leadership process. *Journal of Business Ethics*, 107, 35-47.

Deng, Z. (2016). *Comparative Study of Servant Leadership and Paternalistic Leadership on Employee’s Performance*. Proceedings of the Joint International Conference on Service Science, Management and Engineering and International Conference on Information Science and Technology.

Di Fabio, A., & Peiró, J. (2018). Human Capital Sustainability Leadership to promote sustainable development and healthy organizations: A new scale. *Sustainability*, 10(7), 2413.

Ding, D., Lu, H., Song, Y., & Lu, Q. (2012). Relationship of servant leadership and employee loyalty: The mediating role of employee satisfaction. *iBusiness*, 4(3), 208-215.

Drury, S. (2004). *Employee perceptions of servant leadership: Comparisons by level and with job satisfaction and organizational commitment*. Unpublished doctoral dissertation, Regent University.

Dust, S. B., Resick, C. J., Margolis, J. A., Mawritz, M. B., & Greenbaum, R. L. (2018). Ethical leadership and employee success: Examining the roles of psychological empowerment and emotional exhaustion. *The Leadership Quarterly*, 29(5), 570-583.

Ebrahim, A. B. (2017). *Authentic leadership, trust and work engagement amongst health care workers.* Unpublished doctoral dissertation, North-West University.

Ehrhart, M. G. (2004). Leadership and procedural justice climate as antecedents of unit‐level organizational citizenship behavior. *Personnel psychology*, 57(1), 61-94.

Einstein, J. (2013). *Ethical leadership and service climate: The relationship with job satisfaction and organizational identification.* Unpublished doctoral dissertation, Nova Southeastern University.

Eisenbeiss, S., & Van Knippenberg, D. (2015). On ethical leadership impact: The role of follower mindfulness and moral emotions. *Journal of Organizational Behavior,* 36(2), 182-195.

Eisenbeiss, S., Van Knippenberg, D., & Fahrbach, C. (2015). Doing Well by Doing Good? Analyzing the Relationship Between CEO Ethical Leadership and Firm Performance. *Journal of Business Ethics,* 128(3), 635-651.

Elçi, M., Sener, I., Aksoy, S., & Alpkan, L. (2012). The impact of ethical leadership and leadership effectiveness on employees’ turnover intention: The mediating role of work related stress. *Procedia-Social and Behavioral Sciences,* 58(12)*,* 289-297.

Emuwa, A. (2013). Authentic leadership: Commitment to supervisor, follower empowerment, and procedural justice climate. *Emerging Leadership Journeys*, 6(1), 45-65.

English, E. M. (2011). *Principals' servant leadership and teachers' job satisfaction*. Unpublished doctoral dissertation, University of La Verne.

Erickson, E. L. (2013). *When in the Service of Your Fellow Being: A Mixed Method Study of Servant Leadership and Job Satisfaction in a State Government Setting*. Unpublished doctoral dissertation, Grand Canyon University.

Erkutlu, H., & Chafra, J. (2013). Effects of trust and psychological contract violation on authentic leadership and organizational deviance. *Management Research Review*, 36(9), 828-848.

Erkutlu, H., & Chafra, J. (2015). The effects of empowerment role identity and creative role identity on servant leadership and employees’ innovation implementation behavior. *Procedia - Social and Behavioral Sciences,* 181, 3-11.

Ertureten, A., Cemalcilar, Z., & Aycan, Z. (2013). The relationship of downward mobbing with leadership style and organizational attitudes. *Journal of Business Ethics*, 116(1), 205-216.

Eslamieh, F., & Davoudi, A. H. M. (2016). An analysis of the relationship between managers' ethical leadership style with teachers' organizational commitment and job burnout. *International Journal of Organizational Leadership*, 5(4), 380-392.

Esteinmann, B., Enübold A., & Maier, G. W. (2016). Validation of a German version of the Ethical Leadership at Work questionnaire by Kalshoven and colleagues (2011). *Frontiers in Psychology,* 7, 446.

Eva, N., Newman, A., Miao, Q., Wang, D., & Cooper, B. (2018). Antecedents of Duty Orientation and Follower Work Behavior: The Interactive Effects of Perceived Organizational Support and Ethical Leadership. *Journal of Business Ethics*, in press.

Fallatah, F., Laschinger, H. K., & Read, E. A. (2017). The effects of authentic leadership, organizational identification, and occupational coping self-efficacy on new graduate nurses' job turnover intentions in Canada. *Nursing Outlook*, 65(2), 172-183.

Farooq, H. Q., Salman, M., & Rizwan, M. (2016). The Impact of Ethical Leadership, Leadership Effectiveness, Work Related Stress and Turnover Intention on the Organizational Commitment. *International Journal of Economics and Business Administration*, 2(2), 7-14.

Fox Jr, J. T. (2011). *The impact of personal and organizational identification on the process of teacher trust development within the context of principal authentic leadership*. Unpublished doctoral dissertation, University of Maryland Eastern Shore.

Freij, A., & Olsson, J. (2014). How does managerial leadership influence organizational *ambidexterity?: a study of the public sector*. Unpublished independent thesis, Kristianstad University.

Fuller, B., Bajaba, A., & Bajaba, S. (2018). *A comparison of inspirational and values-based leadership styles as they relate to subordinate curiosity*. Unpublished study.

Galanou, A., & Farrag, D. (2015). Towards the distinctive Islamic mode of leadership in business. *The Journal of Management Development,* 34(8), 882-900.

Gao, Y., & He, W. (2017). Corporate social responsibility and employee organizational citizenship behavior: the pivotal roles of ethical leadership and organizational justice. *Management Decision*, 55(2), 294-309.

Ghahroodi, H. K., Mohd, M. Z. B. T. S., & Ghorban, Z. S. (2013). Examining ethical leadership and its impacts on the followers’ behavioral outcomes. *Asian Social Science,* 9, 91-96.

Giessner, S. R., Kollee, J., van Gils, S., & Van Quaquebeke, N. (2013). *The Interactive Effects of Leader and Follower Moral Identity on Ethical Leadership and LMX Quality*. Proceedings of the Academy of Management Annual Meeting. Briarcliff Manor, NY 10510: Academy of Management.

Goh, S. K., & Zhen-Jie, L. B. (2014). The influence of servant leadership towards organizational commitment: The mediating role of trust in leaders. *International Journal of Business and Management*, 9(1), 17-25.

Gok, K., Sumanth, J. J., Bommer, W. H., Demirtas, O., Arslan, A., Eberhard, J., Ozdemir, A., & Yigit, A. (2017). You may not reap what you sow: How employees’ moral awareness minimizes ethical leadership’s positive impact on workplace deviance. *Journal of Business Ethics*, 146(2), 257-277.

Green, A. E., Miller, E. A., & Aarons, G. A. (2013). Transformational leadership moderates the relationship between emotional exhaustion and turnover intention among community mental health providers. *Community Mental Health Journal*, 49(4), 373-379.

Grobler, A., & Holtzhausen, M. M. (2018). Supervisory trust to be earned: The role of ethical leadership mediated by person-organisational fit. *South African Journal of Economic and Management Sciences*, 21(1), 1-11.

Gucel, C., Tokmak, I., & Turgut, H. (2012). The relationship of the ethical leadership among the organizational trust, affective commitment, and job satisfaction: Case study of a university. *International Journal of Social Sciences and Humanity Studies,* 4(2), 101-110.

Gul, S., Ahmad, B., Rehman, S. U., Shabir, N., & Razzaq, N. (2012). Leadership styles, turnover intentions and the mediating role of organisational commitment. *Information and Knowledge Management*, 2(7), 44-51.

Güngör, S. K. (2016). The Prediction Power of Servant and Ethical Leadership Behaviours of Administrators on Teachers' Job Satisfaction. *Universal Journal of Educational Research*, 4(5), 1180-1188.

Gunnarsdóttir, S. (2014). Is servant leadership useful for sustainable Nordic health care? *Vård i Norden*, 34(2), 53-55.

Hamstra, M. R., Van Yperen, N. W., Wisse, B., & Sassenberg, K. (2011). Transformational-transactional leadership styles and followers’ regulatory focus. *Journal of Personnel Psychology*, 10, 182-186.

Han, R. (2014). Hotel employees' perceptions of supervisors' servant leadership behaviors, and relationships with employees' affective commitment. Unpublished Master’s thesis, Iowa State University.

Han, S. S., & Kim, N. E. (2012). Effects of nurses' perception of servant leadership on leader effectiveness, satisfaction and additional effort: Focused on the mediating effects of leader trust and value congruence. *Journal of Korean Academy of Nursing*, 42(1), 85-94.

Hannah, S., Jennings, P., Bluhm, D., Peng, A., & Schaubroeck, J. (2014). Duty orientation: Theoretical development and preliminary construct testing. *Organizational Behavior and Human Decision Processes,* 123, 220-238.

Hanse, J. J., Harlin, U., Jarebrant, C., Ulin, K., & Winkel, J. (2016). The impact of servant leadership dimensions on leader–member exchange among health care professionals. *Journal of Nursing Management*, 24(2), 228-234.

Hansen, S. D. (2010). *When and how does ethical leadership impact important organizational outcomes? A multi-foci social exchange perspective*. Unpublished doctoral dissertation, Purdue University.

Hansen, S. D., Alge, B. J., Brown, M. E., Jackson, C. L., & Dunford, B. B. (2013). Ethical leadership: Assessing the value of a multifoci social exchange perspective. *Journal of Business Ethics*, 115(3), 435-449.

Harvey, P., Harris, K. J., Kacmar, K. M., Buckless, A., & Pescosolido, A. T. (2014). The impact of political skill on employees’ perceptions of ethical leadership. *Journal of Leadership and Organizational Studies*, 21(1), 5-16.

Hassan, A., & Ahmed, F. (2011). Authentic leadership, trust and work engagement. *International Journal of Human and Social Sciences*, 6(3), 164-170.

Hassan, S, Mashud, R., Yukl, G., & Prussia, G. E. (2012). Ethical and empowering leadership and leader effectiveness. *Journal of Managerial Psychology,* 28*,* 133-146.

Henderson, L. M. (2013). *Enhancing safety communication with leadership: A quantitative study of leadership style and safety voice*. Unpublished doctoral dissertation, University of Phoenix.

Hendler, D. (2011). *Psychological safety as a mediating variable in the relationship between ethical leadership and employee engagement at work.* Unpublished Master’s thesis, University of the Wisrand.

Herman, H. M., Huang, X., & Lam, W. (2013). Why does transformational leadership matter for employee turnover? A multi-foci social exchange perspective. *The Leadership Quarterly*, 24(5), 763-776.

Hoi, J. H. (2018). Ethical Leadership, Ethical Climate, Unethical Behavior, Organizational Commitment in Physical Education. *Digital Fusion Research*, 16(7), 451-460.

Huang, J., Li, W., Qiu, C., Yim, F., & Wan, J. (2016). The impact of CEO servant leadership on firm performance in the hospitality industry. *International Journal of Contemporary Hospitality Management,* 28(5), 945-968.

Hughes, L. W., Avey, J. B., & Nixon, D. R. (2010). Relationships between leadership and followers’ quitting intentions and job search behaviors. *Journal of Leadership and Organizational Studies*, 17(4), 351-362.

Huhtala, M., Kangas, M., Lämsä, A. M., & Feldt, T. (2013). Ethical managers in ethical organisations? The leadership-culture connection among Finnish managers. *Leadership and Organization Development Journal*, 34(3), 250-270.

Hunter, E. M., Neubert, M. J., Perry, S. J., Witt, L. A., Penney, L. M., & Weinberger, E. (2013). Servant leaders inspire servant followers: Antecedents and outcomes for employees and the organization. *The Leadership Quarterly*, 24(2), 316-331.

Inbarasu, J. (2008). *Influence of servant-leadership practice on job satisfaction: A correlational study in a Lutheran organization*. Unpublished doctoral dissertation, University of Phoenix.

Irving, J. A. (2005). Servant leadership and the effectiveness of teams. Unpublished doctoral dissertation, Regent University.

Irving, J. A., & Berndt, J. (2017). Leader Purposefulness within Servant Leadership: Examining the Effect of Servant Leadership, Leader Follower-Focus, Leader Goal-Orientation, and Leader Purposefulness in a Large US Healthcare Organization. *Administrative Sciences*, 7(2), 10-20.

Ismail, I. B., & Daud, Y. (2014). Teacher’s job satisfaction as a mediator of the relationship between ethical leadership and organizational commitment in school. *International Journal of Scientific Research and Education*, 2*,* 1728-1740.

Jaiswal, N. K., & Dhar, R. L. (2017). The influence of servant leadership, trust in leader and thriving on employee creativity. *Leadership and Organization Development Journal*, 38(1), 2-21.

Jang, J., & Kandampully, J. (2018). Reducing employee turnover intention through servant leadership in the restaurant context: A mediation study of affective organizational commitment. *International Journal of Hospitality and Tourism Administration*, 19(2), 125-141.

Jaramillo, F., Bande, B., & Varela, J. (2015). Servant leadership and ethics: A dyadic examination of supervisor behaviors and salesperson perceptions. *Journal of Personal Selling and Sales Management,* 35(2), 108-124.

Jaramillo, F., Grisaffe, D. B., Chonko, L. B., & Roberts, J. A. (2009). Examining the impact of servant leadership on salesperson’s turnover intention. *Journal of Personal Selling and Sales Management*, 29(4), 351-365.

Javed, B., Rawwas, M. Y., Khandai, S., Shahid, K., & Tayyeb, H. H. (2018). Ethical leadership, trust in leader and creativity: The mediated mechanism and an interacting effect. *Journal of Management and Organization*, 24(3), 388-405.

Johnson, H. (2016). *Servant leadership in intercollegiate athletics: Follower perceptions of NCAA division II athletic directors*. Proceedings of the North American Society for Sport Management Conference.

Joo, B. K., & Nimon, K. (2014). Two of a kind? A canonical correlational study of transformational leadership and authentic leadership. *European Journal of Training and Development*, 38(6), 570-587.

Jordan, M. K. (2015). *Determining the relationship between servant leadership and job satisfaction among U.S. navy personnel.* Unpublished doctoral dissertation, Northcentral University.

Joseph, E. E., & Winston, B. E. (2005). A correlation of servant leadership, leader trust, and organizational trust. *Leadership and Organization Development Journal*, 26(1), 6-22.

Kacmar, K. M., andrews, M. C., Harris, K. J., & Tepper, B. J. (2013). Ethical leadership and subordinate outcomes: The mediating role of organizational politics and the moderating role of political skill. *Journal of Business Ethics,* 115*,* 33-44.

Kacmar, K. M., Bachrach, D. G., Harris, K. J., & Zivnuska, S. (2011). Fostering good citizenship through ethical leadership: Exploring the moderating role of gender and organizational politics. *Journal of Applied Psychology,* 96, 633-642.

Kacmar, K. M., Carlson, D. S., & Harris, K. J. (2013). Interactive effect of leaders' influence tactics and ethical leadership on work effort and helping behavior. *The Journal of Social Psychology,* 153*,* 577-597.

Kalshoven, K., & Boon, C. T. (2012). Ethical Leadership, Employee Well-Being, and Helping. *Journal of Personnel Psychology,* 11, 60-68.

Kalshoven, K., & Den Hartog, D. N. (2009). Ethical leader behavior and leader effectiveness: The role of prototypicality and trust. *International Journal of Leadership Studies*, 5(2), 102-120.

Kalshoven, K., Den Hartog, D. N., & De Hoogh, A. H. (2011). Ethical leadership at work questionnaire (ELW): Development and validation of a multidimensional measure. *The Leadership Quarterly*, 22(1), 51-69.

Kang, S. W., Park, H. J., & Jung, D. (2013). *Why does ethical leadership matter in workplace? Empirical evidence from South Korea.* Proceedings of the Academy of Management Annual Meeting, Orlando, FL.

Karami, M., Khan, S., Saddique, M., & Joharishirazi, M. (2013). The job turnover, job satisfaction, ethical leadership and organizational commitment impact on organizational citizenship behaviour. *Journal of Applied Science and Agriculture,* 8*,* 607-611.

Kashyap, V., & Rangnekar, S. (2016). Servant leadership, employer brand perception, trust in leaders and turnover intentions: a sequential mediation model. *Review of Managerial Science*, 10(3), 437-461.

Khokhar, A. M., & Zia-ur-Rehman, M. (2017). Linking Ethical Leadership to Employees' Performance: Mediating Role of Organizational Citizenship Behavior and Counterproductive Work Behavior. *Pakistan Journal of Commerce and Social Sciences*, 11(1), 321-350.

Khuong, M. N., & Yen, N. H. (2014). The effects of leadership styles and sociability trait emotional intelligence on employee engagement – A study in Binh Duong City, Vietnam. *International Journal of Current Research and Academic Review,* 2*,* 121-136.

Kiersch, C. E. (2012). *A multi-level examination of authentic leadership and organizational justice in uncertain times.* Unpublished doctoral dissertation, Colorado State University.

Kottke, J. L., & Pelletier, K. L. (2013). Measuring and differentiating perceptions of supervisor and top leader ethics. *Journal of Business Ethics,* 113*,* 415-428.

Kwak, W., & Kim, H. (2015). Servant leadership and customer service quality at Korean hotels: Multilevel organizational citizenship behavior as a mediator. *Social Behavior and Personality,* 43(8), 1287-1298.

Lagan, T. E. (2007). *Examining authentic leadership: Development of a four-dimensional scale and identification of a nomological network*. Unpublished doctoral dissertation, State University of New York at Albany.

Lapointe, É., & Vandenberghe, C. (2018). Examination of the relationships between servant leadership, organizational commitment, and voice and antisocial behaviors. *Journal of Business Ethics*, 148(1), 99-115.

Laschinger, H. K. S., & Fida, R. (2014). A time-lagged analysis of the effect of authentic leadership on workplace bullying, burnout, and occupational turnover intentions. *European Journal of Work and Organizational Psychology*, 23(5), 739-753.

Laschinger, H. K. S., Wong, C. A., & Grau, A. L. (2012). The influence of authentic leadership on newly graduated nurses’ experiences of workplace bullying, burnout and retention outcomes: A cross-sectional study. *International Journal of Nursing Studies*, 49(10), 1266-1276.

Laschinger, H. K. S., Wong, C. A., & Grau, A. L. (2013). Authentic leadership, empowerment and burnout: a comparison in new graduates and experienced nurses. *Journal of Nursing Management*, 21(3), 541-552.

Laschinger, H. K., & Smith, L. M. (2013). The influence of authentic leadership and empowerment on new-graduate nurses’ perceptions of interprofessional collaboration. *Journal of Nursing Administration*, 43(1), 24-29.

Laub, J. A. (1999). *Assessing the servant organization: Development of the servant. Organizational leadership assessment (SOLA) instrument*. Unpublished doctoral dissertation, Florida Atlantic University.

Lavoie‐Tremblay, M., Fernet, C., Lavigne, G. L., & Austin, S. (2016). Transformational and abusive leadership practices: Impacts on novice nurses, quality of care and intention to leave. *Journal of Advanced Nursing*, 72(3), 582-592.

Lee, J. H., & Cha, J. I. (2015). Effects of Authentic Leadership on Subordinates' Job Performance and Turnover Intention: Subordinates' Organizational Identification as a Mediating Role. *The Journal of the Korea Contents Association*, 15(12), 421-435.

Lee, K. (2016). Ethical leadership and followers' taking charge: Trust in, and identification with, leader as mediators. *Social Behavior and Personality: An International Journal*, 44(11), 1793-1802.

Lelchook, A. M. (2012). *Antecedents and outcomes of workplace engagement.* Unpublished doctoral dissertation, Wayne State University.

Letwin, C., Wo, D., Folger, R., Rice, D., Taylor, R., Richard, B., & Taylor, S. (2016). The "Right" and the "Good" in Ethical Leadership: Implications for Supervisors' Performance and Promotability Evaluations. *Journal of Business Ethics,* 137(4), 743-755.

Lewis, T. M. (2010). *The influence of authenticity and emotional intelligence on the relationship between self-monitoring and leadership effectiveness.* Unpublished doctoral dissertation, Alliant International University.

Li, C., Wu, K., Johnson, D., & Avey, J. (2017). Going Against the Grain Works: An Attributional Perspective of Perceived Ethical Leadership. *Journal of Business Ethics,* 141(1), 87-102.

Liden, R. C., Wayne, S. J., Liao, C., & Meuser, J. D. (2014). Servant leadership and serving culture: Influence on individual and unit performance. *Academy of Management Journal*, 57(5), 1434-1452.

Liden, R. C., Wayne, S. J., Meuser, J. D., Hu, J., Wu, J., & Liao, C. (2015). Servant leadership: Validation of a short form of the SL-28. *The Leadership Quarterly,* 26(2), 254-269.

Liden, R. C., Wayne, S. J., Zhao, H., & Henderson, D. (2008). Servant leadership: Development of a multidimensional measure and multi-level assessment. *The Leadership Quarterly*, 19(2), 161-177.

Lin, C. P., & Liu, M. L. (2017). Examining the effects of corporate social responsibility and ethical leadership on turnover intention. *Personnel Review*, 46(3), 526-550.

Ling, Q., Liu, F., & Wu, X. (2016). Servant Versus Authentic Leadership. *Cornell Hospitality Quarterly,* 58(1), 53-68.

Liu, F., He, S., Tang, Y., & Liu, X. (2014). *The impact of authentic leadership on employees' work engagement: A multilevel study in Chinese hospitality industry*. Proceedings of the 11th International Conference on Service Systems and Service Management (ICSSSM; pp. 1-6). IEEE.

Liu, J., Kwan, HK, Fu, P., & Mao, Y. (2013). Ethical leadership and job performance in China: The roles of workplace friendships and traditionality. *Journal of Occupational and Organizational Psychology,* 86, 564-584.

Liu, Y. (2012). *Linking authentic leadership to positive employee health, behavioral engagement, and job performance*. Unpublished doctoral dissertation, Louisiana Tech University.

Liu, Y., Loi, R., & Lam, L. W. (2013). Exemplification and Supervisor‐rated Performance: The moderating role of ethical leadership. *International Journal of Selection and Assessment*, 21(2), 145-154.

Long, C. S., Thean, L. Y., Ismail, W. K. W., & Jusoh, A. (2012). Leadership styles and employees’ turnover intention: Exploratory study of academic staff in a Malaysian College. *World Applied Sciences Journal*, 19(4), 575-581.

Lu, C., & Lin, C. (2014). The effects of ethical leadership and ethical climate on employee ethical behavior in the international port complex. *Journal of Business Ethics,* 124, 209-223.

Lu, J., Zhang, Z., & Jia, M. (2018). Does Servant Leadership Affect Employees’ Emotional Labor? A Social Information-Processing Perspective. *Journal of Business Ethics*, 159(2), 507-518.

Lu, X. (2014). Ethical leadership and organizational citizenship behavior: The mediating roles of cognitive and affective trust. *Social Behavior and Personality: An International Journal*, 42(3), 379-389.

Lu, X., & Guy, M. E. (2014). How emotional labor and ethical leadership affect job engagement for Chinese public servants. *Public Personnel Management*, 43(1), 3-24.

Lux, A. A. (2012). *Followers perceptions of authentic leadership and their job satisfaction: Impact of ethnicity*. Unpublished Master’s thesis, Auckland University of Technology.

Mahsud, R., Yukl, G., & Prussia, G. (2010). Leader empathy, ethical leadership, and relations-oriented behaviors as antecedents of leader-member exchange quality**.** *Journal of Managerial Psychology,* 25, 561-577.

Majeed, N., Jamshed, S., & Mustamil, N. M. (2018). Striving to restrain employee turnover intention through ethical leadership and pro-social rule breaking. *International Online Journal of Educational Leadership*, 2(1), 39-53.

Malik, M., Munir, Y., Javaid, B., Arshad, A., Nawaz, R., & Nazir, S. (2013). Empirical investigation of indicators of organizational learning: Evidence from Water and Power Development Authority (WAPSA) Pakistan. *Far East Journal of Psychology and Business,* 10, 25-32.

Malingumu, W., Stouten, J., Euwema, M., & Babyegeya, E. (2016). Servant leadership, organisational citizenship behavior and creativity: The mediating role of team-member exchange. *Psychologica Belgica*, 56(4), 342-356.

Mayer, D. M., Bardes, M., & Piccolo, R. F. (2008). Do servant-leaders help satisfy follower needs? An organizational justice perspective. *European Journal of Work and Organizational Psychology*, 17(2), 180-197.

Mayer, D. M., Kuenzi, M., & Greenbaum, R. L. (2010). Examining the link between ethical leadership and employee misconduct: The mediating role of ethical climate. *Journal of Business Ethics*, 95(1), 7-16.

Mazarei, E., Hoshyar, M., & Nourbakhsh, P. (2013). The relationships between servant leadership style and organizational commitment. *Archives of Applied Science Research*, 5(1), 312-317.

McCann, J. T., Graves, D., & Cox, L. (2014). Servant leadership, employee satisfaction, and organizational performance in rural community hospitals. *International journal of Business and management*, 9(10), 28-38.

Mehmood, S. (2016). Impact of ethical leadership on employee creativity: Mediating role of trust and moderating role of creative self-efficacy. *Jinnah Business Review*, 4(2), 65-74.

Men, L. (2012). *The effects of organizational leadership on strategic internal communication and employee outcomes*. Unpublished doctoral dissertation, University of Miami.

Mete, B., & Demir, H. (2016). The effect of ethical leadership on loyalty to supervisor: The mediator role of trust*. Faculty of Economics and Administrative Sciences Journal*, 7(14), 557-576.

Miao, Q., Newman, A., Yu, J., & Xu, L. (2013). The relationship between ethical leadership and unethical pro-organizational behavior: Linear or curvilinear effects? *Journal of Business Ethics,* 116, 641-653.

Min, J. 2016. Influence of head nurses' ethical leadership on job satisfaction among staff nurses: Mediating effect of affective commitment. *Journal of Korean Academic Nursing*, 22(5), 553-561.

Miniotaitė, A. (2012). *The impact of authentic leadership on follower self-actualization*. Unpublished doctoral dissertation, ISM University of Management and Economics.

Mitterer, D. M. (2017). *Servant leadership and its effect on employee job satisfaction and turnover intent.* Unpublished doctoral dissertation, Walden University.

Mo, S., & Shi, J. (2017). Linking ethical leadership to employee burnout, workplace deviance and performance: Testing the mediating roles of trust in leader and surface acting. *Journal of Business Ethics*, 144(2), 293-303.

Mo, S., Wang, Z., Akrivou, K., & Booth, S. A. (2012). Look up, look around: Is there anything different about team-level OCB in China? *Journal of Management and Organization*, 18(6), 818-832.

Mol, E. (2011). *The values-based organization: How ethical leadership affects work-related outcomes*. Unpublished Master’s thesis, University of Mastricht.

Moon, K.-K., & Jung, C. (2018). Management Representativeness, Ethical Leadership, and Employee Job Satisfaction in the U.S. Federal Government. *Public Personnel Management*, 47(3), 265–286.

Mostafa, A. M. S. (2018). Ethical leadership and organizational citizenship behaviours: The moderating role of organizational identification. *European Journal of Work and Organizational Psychology*, 27(4), 441-449.

Mozumder, N. A. (2018). A multilevel trust-based model of ethical public leadership. *Journal of Business Ethics*, 153(1), 167-184.

Mulligan, D. (2016). *Servant leadership and its impact on classroom climate and student achievement*. Unpublished doctoral dissertation, Grand Canyon University.

Muthia, A., & Krishnan, V. R. (2015). Servant leadership and commitment: Role of transformational leadership. *International Journal on Leadership*, 3(1), 9-20.

Neider, L. L., & Schriesheim, C. A. (2011). The authentic leadership inventory (ALI): Development and empirical tests. *The Leadership Quarterly*, 22(6), 1146-1164.

Neubert, Hunter, & Tolentino. (2016). A servant leader and their stakeholders: When does organizational structure enhance a leader's influence? *The Leadership Quarterly,* 27(6), 896-910.

Neubert, M. J., Carlson, D. S., Kacmar, K. M., Roberts, J. A., & Chonko, L. B. (2009). The virtuous influence of ethical leadership behavior: Evidence from the field. *Journal of Business Ethics,* 90, 157-170.

Neubert, M. J., Kacmar, K. M., Carlson, D. S., Chonko, L. B., & Roberts, J. A. (2008). Regulatory focus as a mediator of the influence of initiating structure and servant leadership on employee behavior. *Journal of Applied Psychology*, 93(6), 1220-1233.

Neubert, M. J., Wu, C., & Roberts, J. A. (2013). The influence of ethical leadership and regulatory focus on employee outcomes. *Business Ethics Quarterly,* 23, 269-296.

Neves, P., & Story, J. (2015). Ethical Leadership and Reputation: Combined Indirect Effects on Organizational Deviance. *Journal of Business Ethics,* 127(1), 165-176.

Newman, A., Kiazad, K., Miao, Q., & Cooper, B. (2014). Examining the cognitive and affective trust-based mechanisms underlying the relationship between ethical leadership and organisational citizenship: A case of the head leading the heart? *Journal of Business Ethics*, 123(1), 113-123.

Newman, A., Schwarz, G., Cooper, B., & Sendjaya, S. (2017). How servant leadership influences organizational citizenship behavior: The roles of LMX, empowerment, and proactive personality. *Journal of Business Ethics,* 145(1), 49-62.

Ng, T., & Feldman, D. (2015). Ethical leadership: Meta-analytic evidence of criterion-related and incremental validity. *Journal of Applied Psychology,* 100(3), 948-965.

Nguyen, B. (2015). *The effect of ethical leadership, behavioural integrity, and moral disengagement in predicting turnover intentions during newcomer socialization.* Unpublished doctoral dissertation, University of Calgary.

Nielsen, M. B. (2013). Bullying in work groups: The impact of leadership. *Scandinavian Journal of Psychology*, 54(2), 127-136.

Nobari, E., Mohamadkhani, K., & Mohammad Davoudi, A. (2014). The relationship between servant leadership and organizational citizenship behavior of employees at valiasr academic complex, islamic azad university-central tehran branch. International Journal of Management and Business Research, 4(4), 247-254.

Norman, S. M. (2006). The role of trust: Implications for psychological capital and authentic leadership. Unpublished doctoral dissertation, University of Nebraska.

Ogunfowora, B. (2009). *The consequences of ethical leadership: Comparisons with transformational leadership and abusive supervision.* Unpublished thesis, University of Calgary.

Ogunfowora, B. (2014). It’s all a matter of consensus: Leader role modeling strength as a moderator of the links between ethical leadership and employee outcomes. *Human Relations*, 67(12), 1467-1490.

Olaniyan, O. S., & Hystad, S. W. (2016). Employees’ psychological capital, job satisfaction, insecurity, and intentions to quit: The direct and indirect effects of authentic leadership. *Revista de Psicología del Trabajo y de las Organizaciones*, 32(3), 163-171.

Onorato, M. (2013). An empirical study of unethical leadership and workplace bullying in industry segments. *SAM Advanced Management Journal*, 78(2), 4-16.

Ostrem, L. M. (2006). *Servant leadership and work-related outcomes: A multilevel model*. Unpublished doctoral dissertation, University of Nebraska-Lincoln.

Palanski, M., Avey, J. B., & Jiraporn, N. (2014). The effects of ethical leadership and abusive supervision on job search behaviors in the turnover process. *Journal of Business Ethics,* 121*,* 135-146.

Palmer, N. (2013). *The effects of leader behavior on follower ethical behavior: Examining the mediating roles of ethical efficacy and moral disengagement*. Unpublished doctoral dissertation. University of Nebraska.

Panaccio, A., Henderson, D. J., Liden, R. C., Wayne, S. J., & Cao, X. (2015). Toward an understanding of when and why servant leadership accounts for employee extra-role behaviors. *Journal of Business and Psychology*, 30(4), 657-675.

Pavese-Kaplan, E. P. (2013). *The influence of perceived ethical culture and ethical leadership on job and organizational engagement.* Unpublished doctoral dissertation, Seattle Pacific University.

Pei-Ju Chuang & Su-Fen Chiu (2016): When Moral Personality and Moral Ideology Meet Ethical Leadership: A Three-Way Interaction Model. *Ethics and Behavior*, 28, 45-69.

Peng, J. C., Jien, J. J., & Lin, J. (2018). Antecedents and consequences of psychological contract breach. *Journal of Managerial Psychology*, 31(8), 1312-1326.

Persaud, D. (2015). *A correlational study of servant leadership and employee job satisfaction in New York city public hospital emergency rooms.* Unpublished doctoral dissertation, Grand Canyon University.

Peterson, S. J., Galvin, B. M., & Lange, D. (2012). CEO servant leadership: Exploring executive characteristics and firm performance. *Personnel Psychology*, 65(3), 565-596.

Philipp, B. L. (2012). *Psychological contracts in the workplace: Relationships among organizational commitment, organizational citizenship behaviors, and ethical leadership.* Unpublished doctoral dissertation, Alliant International University.

Philipp, B. L., & Lopez, P. D. J. (2013). The moderating role of ethical leadership: Investigating relationships among employee psychological contracts, commitment, and citizenship behavior. *Journal of Leadership and Organizational Studies,* 20, 304-315.

Piccolo, R. F., Greenbaum, R., Hartog, D. N. D., & Folger, R. (2010). The relationship between ethical leadership and core job characteristics. *Journal of Organizational Behavior,* 31, 259-278.

Politis, J. (2013). *The relationship between team performance, authentic and servant leadership*. Proceedings of the European Conference on Management, Leadership and Governance (pp. 237-244).

Ponnu, C. H., & Tennakoon, G. (2009). The association between ethical leadership and employee outcomes – the Malaysian Case. *Electronic Journal of Business Ethics and Organization Studies,* 14*,* 21-32.

Potipiroon, W., & Ford, M. T. (2014). *When Does Public Service Motivation Relate to Organizational Commitment? A Three-Way Interaction*. Proceedings of the Academy of Management Annual Meeting (Vol. 2014, No. 1, pp. 10948). Briarcliff Manor, NY 10510: Academy of Management.

Pucic, J. (2011). *Ethical leadership in the employment relationship: Evidence from three Canadian surveys.* Unpublished doctoral dissertation, University of Toronto.

Qi, Y., & Ming-Xia, L. (2014). Ethical leadership, organizational identification and employee voice: Examining moderated mediation process in the Chinese insurance industry. *Asia Pacific Business Review*, 20(2), 231-248.

Qian, J., Lin, X., & Chen, G. Z. X. (2012). Authentic leadership and feedback-seeking behaviour: An examination of the cultural context of mediating processes in China. *Journal of Management and Organization*, 18(3), 286-299.

Qin, Q., Wen, B., Ling, Q., Zhou, S., & Tong, M. (2014). How and when the effect of ethical leadership occurs? A multilevel analysis in the Chinese hospitality industry. *International Journal of Contemporary Hospitality Management*, 26(6), 974-1001.

Quade, M. J., Perry, S. J., & Hunter, E. M. (2019). Boundary conditions of ethical leadership: Exploring supervisor-induced and job hindrance stress as potential inhibitors. *Journal of Business Ethics*, 158(4), 1165-1184.

Quenson, Y. (2013). *The relationship ethical leadership and individual-directed organizational citizenship behaviour: The moderating effect of leader-member-exchange.* Unpublished honors degree thesis, Hong Kong Baptist University.

Ram, P., & Prabhakar, G. V. (2010). Leadership styles and perceived organizational politics as predictors of work related outcomes. *European Journal of Social Sciences*, 15(1), 40-55.

Ramli, A., Desa, N. M., & Ramayah, T. (2014). Servant leadership and organizational citizenship behaviour: The Malaysian perspectives. In *Conference of University Forum for Human Resource Development* (pp. 1-17).

Read, E., & Laschinger, H. K. (2013). Correlates of new graduate nurses’ experiences of workplace mistreatment. *The Journal of Nursing Administration*, 43(4), 221-228.

Rebelo, J. (2012). *Abusive Supervision*. Retrieved from Prezi.com/frhaiplxjwqq/abusive-supervision/.

Reese, J. S. (2017). *Servant leadership in the finance industry: Relation to desired employee outcomes*. Unpublished doctoral dissertation, Capella University.

Reinke, S. J. (2003). Does the form really matter? Leadership, trust, and acceptance of the performance appraisal process. *Review of Public Personnel Administration*, 23(1), 23-37.

Resick, C. J., Hargis, M. B., Shao, P., & Dust, S. B. (2013). Ethical leadership, moral equity judgments, and discretionary workplace behavior. *Human Relations,* 66, 951-972.

Rezaei, M., Salehi, S., Shafiei, M., & Sabet, S. (2012). Servant leadership and organizational trust: The mediating effect of the leader trust and organizational communication. *Emerging Markets Journal*, 2(1), 70-78.

Riggio, R. E., Zhu, W., Reina, C., & Maroosis, J. A. (2010). Virtue-based measurement of ethical leadership: The leadership virtues questionnaire. *Consulting Psychology Journal: Practice and Research,* 62, 235-250.

Rodriguez, J. (2016). *Emotional intelligence and authentic leadership: An empirical exploration of two emerging theoretical constructs*. Unpublished doctoral dissertation, Our Lady of The Lake University.

Rog, E. (2011). *Managers' and Subordinates' Perceptions of Authentic Leadership, Subordinate Outcomes, and Mediating Mechanisms*. Unpublished doctoral dissertation, University of Guelph.

Rog, E. (2011). *Managers' and subordinates' perceptions of authentic leadership, subordinate outcomes, and mediating mechanisms*. Unpublished doctoral dissertation, The University of Guelph.

Roof, R., Winston, Bruce E., Bocarnea, Mihai, & Marshall, J. (2016). *The Relationship Between Spiritual Engagement and Authentic Leadership: Exploring the Core of Leadership*. Unpublished doctoral dissertation, Regent University.

Rose, J. D. (2016). *The effect of American and Nigerian culture on antecedents of ethical leadership*. Unpublished doctoral dissertation, Regent University.

Rouzbahani, M. T., Soleimanian, Z., Rezai, F. B., & Hemati, F. (2013). The relationship between ethical leadership with the three dimensions of organizational commitment (affective, continuous, normative) and confidence in superintendent. *Journal of Basic and Applied Scientific Research,* 3*,* 771-776.

Rubin, R. S., Dierdorff, E. C., & Brown, M. E. (2010). Do ethical leaders get ahead? *Business Ethics Quarterly,* 20, 215-236.

Ruiz‐Palomino, P., Ruiz‐Amaya, C., & Knörr, H. (2011). Employee organizational citizenship behaviour: The direct and indirect impact of ethical leadership. *Canadian Journal of Administrative Sciences*, 28(3), 244-258.

Ruiz, P., Ruiz, C., & Martínez, R. (2011). Improving the “leader–follower” relationship: Top manager or supervisor? The ethical leadership trickle-down effect on follower job response. *Journal of Business Ethics*, 99, 587-608.

Sabir, M. S., Iqbal, J. J., Rehman, K., Shah, K. A., & Yameen, M. (2012). Impact of corporate ethical values on ethical leadership and employee performance. *International Journal of Business and Social Science,* 3*,* 163-171.

Saboe, K. (2010). *Prioritizing those who follow: Servant leadership, needs satisfaction, and positive employee outcomes*. Unpublished doctoral dissertation, University of South Florida, Tampa.

Sağnak, M. (2017). Ethical leadership and teachers’ voice behavior: The mediating roles of ethical culture and psychological safety. *Educational Sciences: Theory and Practice,* 17(4): 973-989.

Salie, A. (2008). *Servant-minded leadership and work satisfaction in Islamic organizations: A correlational mixed study*. Unpublished doctoral dissertation, University of Phoenix.

Schneider, S. K., & George, W. M. (2011). Servant leadership versus transformational leadership in voluntary service organizations. *Leadership and Organization Development Journal*, 32(1), 60-77.

Schuh, S. C., Zhang, X. A., & Tian, P. (2013). For the good or the bad? Interactive effects of transformational leadership with moral and authoritarian leadership behaviors. *Journal of Business Ethics*, 116(3), 629-640.

Schwarz, G., Newman, A., Cooper, B., & Eva, N. (2016). Servant leadership and follower job performance: The mediating effect of public service motivation. *Public Administration*, 94(4), 1025-1041.

Scuderi, N. F. (2010). *Servant leadership and transformational leadership in church organizations*. Unpublished doctoral dissertation, The George Washington University.

Seheult, E. (2016). *A quantitative examination of authentic leadership, tenure, and propensity for knowledge sharing behavior in organizations*. Unpublished doctoral dissertation, Capella University.

Sendjaya, S., & Pekerti, A. (2010). Servant leadership as antecedent of trust in organizations. *Leadership and Organization Development Journal*, 31(7), 643-663.

Sendjaya, S., Eva, N., Butar, I. B., Robin, M., & Castles, S. (2019). SLBS-6: Validation of a Short Form of the Servant Leadership Behavior Scale. *Journal of Business Ethics*, 156, 941-956.

Sepahvand, R., Pirzad, A., & Rastipour, M. (2015). Exploring servant leadership effects on employees satisfaction from work. *International Journal of Asian Social Science*, 5(1), 45-51.

Shaffer, J., DeGeest, D., & Li, A. (2016). Tackling the Problem of Construct Proliferation. *Organizational Research Methods,* 19(1), 80-110.

Shafique, I., N Kalyar, M., & Ahmad, B. (2018). The Nexus of Ethical Leadership, Job Performance, & Turnover Intention: The Mediating Role of Job Satisfaction. *Interdisciplinary Description of Complex Systems*, 16(1), 71-87.

Shao, P. 2010. *Ethics-based leadership & employee ethical behavior: Examining the mediating role of ethical regulatory focus*. Unpublished doctoral dissertation, Drexel University

Shapira-Lishchinsky, O., & Tsemach, S. (2014). Psychological empowerment as a mediator between teachers’ perceptions of authentic leadership & their withdrawal & citizenship behaviors. *Educational Administration Quarterly*, 50(4), 675-712.

Sharif, M. M., & Scandura, T. A. (2012). *Ethical leadership and gratitude during organizational change.* Proceedings of the Academy of Management Annual Meeting, Boston, MA.

Sharif, M. M., & Scandura, T. A. (2014). Do perceptions of ethical conduct matter during organizational change? Ethical leadership and employee involvement. *Journal of Business Ethics*, 124(2), 185-196.

Shim, D., Park, H., & Eom, T. (2016). Public servant leadership: Myth or powerful reality? *International Review of Public Administration,* 21(1), 3-20.

Shin, Y. (2012). CEO ethical leadership, ethical climate, climate strength, and collective organizational citizenship behavior. *Journal of Business Ethics,* 108, 299-312.

Shin, Y., Sung, S., Choi, J., & Kim, M. (2015). Top Management Ethical Leadership and Firm Performance: Mediating Role of Ethical and Procedural Justice Climate. *Journal of Business Ethics,* 129(1), 43-57.

Shor, J., Resick, C. J., & Dust, S. (2013). *Beyond good and bad: Examining the empowering influence of ethical leaders.* Proceedings of the Academy of Management Annual Meeting, Orlando, FL.

Siegel, P. G. (2013). *Ethical leadership and organizational commitment in the Canadian Armed Forces.* Unpublished Master’s thesis, Saint Mary’s University.

Smith, M. B., Koppes Bryan, L., & Vodanovich, S. J. (2012). The counter-intuitive effects of flow on positive leadership and employee attitudes: Incorporating positive psychology into the management of organizations. *The Psychologist-Manager Journal*, 15(3), 174-198.

Sparks, T. E. (2010). *Navigating the leadership labyrinth.* Unpublished Master’s thesis, University of Georgia.

Sparks, T. E. (2012). *Ethical and unethical leadership and followers’ well-being” Exploring psychological processes and boundary conditions.* Unpublished doctoral dissertation, The University of Georgia.

Srivastava, S. (2016). Work deviant behavior-employee engagement: An empirical investigation of the role of ethical leadership of Indian middle level managers. *Drishtikon: A Management Journal,* 7(2), 53-65.

Staab, K. (2016). *Authentic leaders: Differences in character strengths they possess,* Unpublished doctoral dissertation, Gannon University.

Steffens, N. K., Mols, F., Haslam, S. A., & Okimoto, T. G. (2016). True to what We stand for: Championing collective interests as a path to authentic leadership. *The Leadership Quarterly,* 27(5), 726-744.

Stouten, J., Van Dijke, M., Mayer, D. M., De Cremer, D., & Euwema, M. C. (2013). Can a leader be seen as too ethical? The curvilinear effects of ethical leadership. *The Leadership Quarterly,* 24*,* 680-695.

Sutherland Jr., M. A. (2010). *An examination of ethical leadership and organizational commitment.* Unpublished doctoral dissertation, Nova Southeastern University.

Sweet, K. (2012). *A fit model of leadership and two empirical examinations*. Unpublished doctoral dissertation, University of Houston.

Tai, C. L., Chang, C., Hong, J, & Chen, L. (2012). Alternative models for the relationship among leadership, organizational citizenship behavior, and performance: A study of new product development teams in Taiwan. *Procedia – Social and Behavioral Sciences,* 57*,* 511-517.

Tang, G., Cai, Z., Liu, Z., Zhu, H., Yang, X., & Li, J. (2015). The importance of ethical leadership in employees’ value congruence and turnover. *Cornell Hospitality Quarterly*, 56(4), 397-410.

Tanner, C., Brügger, A., van Schie, S., & Lebherz, C. (2010). Actions speak louder than words. *Journal of Psychology,* 218, 225-233.

Tariq, Z., & Ambali, A. R. (2013). Examining servant leadership attributes and employee trust. *Asian Journal of Empirical Research*, 3(5), 551-562.

Tate, B. (2008). A longitudinal study of the relationships among self-monitoring, authentic leadership, and perceptions of leadership. *Journal of Leadership and Organizational Studies*, 15(1), 16-29.

Thun, N. B. (2009). *Character strengths in leadership.* Unpublished doctoral dissertation, Saint Mary’s University.

Tonkin, T. H. (2013). Authentic versus transformational leadership: Assessing their effectiveness on organizational citizenship behavior of followers. *International Journal of Business and Public Administration*, 10(1), 40-61.

Tremblay, M. A. (2010). Fairness perceptions and trust as mediators on the relationship between leadership style, unit commitment, and turnover intentions of Canadian forces personnel. *Military Psychology*, 22(4), 510-523.

Trivers, G. (2009). *Servant leadership's effects on trust and organizational citizenship behaviors.* Unpublished doctoral dissertation, *Argosy University, Seattle, WA*.

Tumasjan, A., Strobel, M., & Welpe, I. (2011). Ethical leadership evaluations after moral transgression: Social distance makes the difference. *Journal of Business Ethics,* 99, 609-622.

Tummers, L., & Knies, E. (2014). *The public leadership questionnaire: The development & validation of five dimensions of public leadership behavior.* Proceedings of the International Research Society for Public Management.

Tuttle, M. D. (2009). *True north or traveled terrain? An empirical investigation of authentic leadership*. Unpublished doctoral dissertation, University of South Florida.

Underwood, R. (2015). *Attachment style, leadership behavior, & perceptions of leader effectiveness in academic management.* Unpublished doctoral dissertation, Walden University.

Van Dierendonck, D., & Nuijten, I. (2011). The servant leadership survey: Development & validation of a multidimensional measure. *Journal of Business & Psychology*, 26(3), 249-267.

Van Dierendonck, D., & Patterson, K. (2010). *Servant leadership: Developments in theory & research*. Springer.

Van Dierendonck, D., Stam, D., Boersma, P., De Windt, N., & Alkema, J. (2014). Same difference? Exploring the differential mechanisms linking servant leadership & transformational leadership to follower outcomes. *The Leadership Quarterly*, 25(3), 544-562.

Van Staden, M. (2007). *The relationship between servant leadership, emotional intelligence, trust in the immediate supervisor & meaning in life: An exploratory study*. Unpublished doctoral dissertation, University of Stellenbosch.

Van Voorst, R. (2012). *A study of the relationships between ethical leadership, leader-member exchange (LMX) & organizational citizenship behavior (OCB).* Unpublished Master’s thesis, Tilburg University.

Velez, M. J., & Neves, P. (2014). *Shaping Emotional Reactions to Ethical Behaviors: Proactive Personality as a Leadership Substitute*. Proceedings of the *Academy of Management* (No. 1, pp. 15618). Briarcliff Manor, NY 10510: Academy of Management.

Verdorfer, A. P., & Peus, C. (2014). The Measurement of servant leadership: Validation of a German version of the servant leadership survey (SLS). Zeitschrift für Arbeitsund Organisationspsychologie *A&O*, 58, 1-16.

Verdorfer, A. P., Steinheider, B., & Burkus, D. (2015). Exploring the socio-moral climate in organizations: An empirical examination of determinants, consequences, & mediating mechanisms. *Journal of Business Ethics*, 132(1), 233-248.

Von Fischer, P. E. (2017). *The Relationship Between Teacher Perceptions of Principal Servant Leadership Behavior & Teacher Job Satisfaction in South Dakota.* Unpublished doctoral dissertation, University of South Dakota.

Waldman, D., Wang, D., Hannah, S., & Balthazard, P. (2017). A neurological and ideological perspective of ethical leadership. *Academy of Management Journal,* *60*(4), 1285-1306.

Walumbwa, F. O., & Lawler, J. J. (2003). Building effective organizations: Transformational leadership, collectivist orientation, work-related attitudes & withdrawal behaviours in three emerging economies. *International Journal of Human Resource Management*, 14(7), 1083-1101.

Walumbwa, F. O., Avolio, B. J., Gardner, W. L., Wernsing, T. S., & Peterson, S. J. (2008). Authentic leadership: Development and validation of a theory-based measure. *Journal of Management*, 34(1), 89-126.

Walumbwa, F. O., Hartnell, C. A., & Oke, A. (2010). Servant leadership, procedural justice climate, service climate, employee attitudes, and organizational citizenship behavior: a cross-level investigation. *Journal of Applied Psychology*, 95(3), 517-529.

Walumbwa, F. O., Mayer, D. M., Wang, P., Wang, H., Workman, K., & Christensen, A. L. (2011). Linking ethical leadership to employee performance: The roles of leader–member exchange, self-efficacy, and organizational identification. *Organizational Behavior and Human Decision Processes,* 115, 204-213.

Walumbwa, F. O., Wang, P., Lawler, J. J., & Shi, K. (2004). The role of collective efficacy in the relations between transformational leadership and work outcomes. *Journal of Occupational and Organizational Psychology*, 77(4), 515-530.

Walumbwa, F. O., Wang, P., Wang, H., Schaubroeck, J., & Avolio, B. J. (2010). Psychological processes linking authentic leadership to follower behaviors. *The Leadership Quarterly*, 21(5), 901-914.

Wang, D. S., & Hsieh, C. C. (2013). The effect of authentic leadership on employee trust and employee engagement. *Social Behavior and Personality: An International Journal*, 41(4), 613-624.

Wang, G., & Hackett, R. (2016). Conceptualization and Measurement of Virtuous Leadership: Doing Well by Doing Good. *Journal of Business Ethics,* 137(2), 321-345.

Washington, R. (2007). *Empirical relationships among servant, transformational, and transactional leadership: Similarities, differences, and correlations with job satisfaction and organizational commitment*. Unpublished doctoral dissertation, Auburn University.

Washington, R. R., Sutton, C. D., & Sauser Jr, W. I. (2014). How distinct is servant leadership theory? Empirical comparisons with competing theories. *Journal of Leadership, Accountability and Ethics*, 11(1), 11-25.

Water, L. (2014). *Effective leadership styles: A pilot study in legal services*. Unpublished Master's thesis, University of Twente.

Water, L. (2014). *Effective leadership styles: a pilot study in legal services*. University of Twente.

Webb, K. J. (2014). *The moderating effect of perceived ethical leadership on reduced audit quality behaviors*. Unpublished doctoral dissertation, University of Texas at Arlington.

West, G. B., Bocârnea, M., & Marañon, D. (2009). Servant-leadership as a predictor of job satisfaction and Organizational commitment with the moderating effects of organizational support and role clarity among Filipino engineering, manufacturing, and technology workers. *The International Journal of Servant-Leadership*, 5(1), 129-162.

Whisnant, B., & Khasawneh, O. (2014). The influence of leadership and trust on the sharing of tacit knowledge: Exploring a path model. *Journal of Business Studies Quarterly*, 6(2), 1-17.

Williams, E. A., Pillai, R., Deptula, B., & Lowe, K. B. (2012). The effects of crisis, cynicism about change, and value congruence on perceptions of authentic leadership and attributed charisma in the 2008 presidential election. *The Leadership Quarterly*, 23(3), 324-341.

Wilson, D. F. (2013). *Servant leadership and job satisfaction in a multicultural hospitality organization: A quantitative, non-experimental descriptive study*. Unpublished doctoral dissertation, Grand Canyon University.

Winston, B., & Fields, D. (2015). Seeking and measuring the essential behaviors of servant leadership. *Leadership and Organization Development Journal,* 36(4), 413-434.

Wolde, A., Groenendaal, J., Helsloot, I., & Schmidt, A. (2014). An explorative study on the effect of ethical leadership on organization misbehavior in a Dutch fire service. *International Journal of Leadership Studies,* 8(2), 18-43.

Wolmarans, J. (2014). *The effect of core ethical values on ethical leadership, organizational justice, ethical climate, and leader effectiveness*. Unpublished Master’s thesis, Stellenbosch University.

Wong, C. A., & Cummings, G. G. (2009). The influence of authentic leadership behaviors on trust and work outcomes of health care staff. *Journal of Leadership Studies*, 3(2), 6-23.

Wong, C. A., & Giallonardo, L. (2013). Authentic leadership and nurse‐assessed adverse patient outcomes. *Journal of Nursing Management*, 21(5), 740-752.

Wong, C. A., & Laschinger, H. K. (2012). Authentic leadership, performance, and job satisfaction: the mediating role of empowerment. *Journal of Advanced Nursing*, 69(4), 947-959.

Wong, C. A., Laschinger, H. K., & Cummings, G. G. (2010). Authentic leadership and nurses' voice behaviour and perceptions of care quality. *Journal of Nursing Management*, 18(8), 889-900.

Wu, L. Z., Kwan, H., Yim, F., Chiu, R., & He, X. (2015). CEO Ethical Leadership and Corporate Social Responsibility: A Moderated Mediation Model. *Journal of Business Ethics,* 130(4), 819-831.

Wu, L. Z., Tse, E. C. Y., Fu, P., Kwan, H. K., & Liu, J. (2013). The impact of servant leadership on hotel employees’ “servant behavior”. *Cornell Hospitality Quarterly*, 54(4), 383-395.

Wu, M. (2012). Moral leadership and work performance: Testing the mediating and interaction effects in China. *Chinese Management Studies*, 6(2), 284-299.

Wu, M., Huang, X., & Chan, S. C. (2012). The influencing mechanisms of paternalistic leadership in Mainland China. *Asia Pacific Business Review*, 18(4), 631-648.

Wu, M., Huang, X., Li, C., & Liu, W. (2012). Perceived interactional justice and trust‐in‐supervisor as mediators for paternalistic leadership. *Management and Organization Review*, 8(1), 97-121.

Xu, A., Loi, R., & Ngo, H. (2016). Ethical Leadership Behavior and Employee Justice Perceptions: The Mediating Role of Trust in Organization. *Journal of Business Ethics,* 134(3), 493-504.

Yang, Q., & Wei, H. (2018). The impact of ethical leadership on organizational citizenship behavior: The moderating role of workplace ostracism. *Leadership and Organization Development Journal*, 39(1), 100-113.

Yates, L. (2014). Exploring the relationship of ethical leadership with job satisfaction, organizational commitment, and organizational citizenship behavior. *The Journal of Values-Based Leadership,* 7(1), 1-15.

Yemi-Sofumade, H. B. (2012). The relationship between ethical and authentic nurse leadership and the turnover intentions of staff nurses. Unpublished doctoral dissertation, Capella University, Minneapolis, MN.

Yidong, T., & Xinxin, L. (2013). How ethical leadership influence employees’ innovative work behavior: A perspective of intrinsic motivation. *Journal of Business Ethics,* 116, 441-455.

Yukl, G., Mahsud, R., Hassan, S., & Prussia, G. E. (2013). An improved measure of ethical leadership. *Journal of Leadership and Organizational Studies,* 20, 38-48.

Yurtkoru, E. S., Ensari, S., & ErdilekKarabay, M. (2018). To what extent trust in leader and ethical climate affect turnover intention? A research on private and public bank employees. *International Journal of Organizational Leadership*, 12-26.

Zafar, A. (2013). *Linking ethical leadership and employees’ in-role performance: Exploring the mediating roles of psychological capital and follower-leader relational capital.* Unpublished Master’s thesis, Brock University.

Zehir, C., & Erdogan, E. (2011). The association between organizational silence and ethical leadership through employee performance. *Procedia-Social and Behavioral Sciences*, 24, 1389-1404.

Zehir, C., Akyuz, B., Eren, M. S., & Turhan, G. (2013). The indirect effects of servant leadership behavior on organizational citizenship behavior and job performance: Organizational justice as a mediator. *International Journal of Research in Business and Social Science*, 2(3), 1-13.

Zhao, C., Liu, Y., & Gao, Z. (2016). An identification perspective of servant leadership's effects. *Journal of Managerial Psychology,* 31(5), 898-913.

Zheng, X., Zhu, W., Yu, H., Zhang, X., & Zhang, L. (2011). Ethical leadership in Chinese organizations: Developing a scale. *Frontiers of Business Research in China*, 5, 179-198.

Zhou, Y., & Miao, Q. (2014). Servant leadership and affective commitment in the Chinese public sector: The mediating role of perceived organizational support. *Psychological Reports*, 115(2), 381-395.

Zhu, W. (2006). *Authentic leadership and follower moral decision intention: Role of follower moral identity*. Unpublished doctoral dissertation, University of Nebraska.

Zhu, W., He, H., Treviño, L. K., Chao, M. M., & Wang, W. (2015). Ethical leadership and follower voice and performance: The role of follower identifications and entity morality beliefs. *The Leadership Quarterly*, 26(5), 702-718.

Zimmerer, T. E. (2013). *Generational perceptions of servant leadership: A mixed methods study*. Unpublished doctoral dissertation, Capella University.

Zoghbi-Manrique-de-Lara, P., & Suarez-Acosta, M. A. (2014). Employees’ reacts to peers’ unfair treatment by supervisors: The role of ethical leadership. *Journal of Business Ethics,* 122*,* 537-549.

Zou, W. C., Tian, Q., & Liu, J. (2015). Servant leadership, social exchange relationships, and follower's helping behavior: Positive reciprocity belief matters. *International Journal of Hospitality Management*, 51, 147-156.
